# Catalytic processing in ruthenium-based polyoxometalate coacervate protocells

**DOI:** 10.1038/s41467-019-13759-1

**Published:** 2020-01-03

**Authors:** Pierangelo Gobbo, Liangfei Tian, B. V. V. S Pavan Kumar, Samuel Turvey, Mattia Cattelan, Avinash J. Patil, Mauro Carraro, Marcella Bonchio, Stephen Mann

**Affiliations:** 1 Centre for Organized Matter Chemistry and Centre for Protolife Research, School of Chemistry, University of Bristol, Bristol, BS8 1TS UK; 2 School of Chemistry, University of Bristol, Bristol, BS8 1TS UK; 3 ITM-CNR and Dipartimento di Scienze Chimiche, Università di Padova, Via F. Marzolo 1, 35131 Padova, Italy

**Keywords:** Biocatalysis, Bioinspired materials, Bioinspired materials

## Abstract

The development of programmable microscale materials with cell-like functions, dynamics and collective behaviour is an important milestone in systems chemistry, soft matter bioengineering and synthetic protobiology. Here, polymer/nucleotide coacervate micro-droplets are reconfigured into membrane-bounded polyoxometalate coacervate vesicles (PCVs) in the presence of a bio-inspired Ru-based polyoxometalate catalyst to produce synzyme protocells (Ru_4_PCVs) with catalase-like activity. We exploit the synthetic protocells for the implementation of multi-compartmentalized cell-like models capable of collective synzyme-mediated buoyancy, parallel catalytic processing in individual horseradish peroxidase-containing Ru_4_PCVs, and chemical signalling in distributed or encapsulated multi-catalytic protocell communities. Our results highlight a new type of catalytic micro-compartment with multi-functional activity and provide a step towards the development of protocell reaction networks.

## Abstract (web summary)

The development of microscale materials with cell-like functions and collective behaviors is an important milestone in bottom-up synthetic biology. Here the authors employ a bio-inspired inorganic synzyme to construct a micro-compartment with multi-functional activity providing a step towards the development of protocell reaction networks.

## Introduction

The endogenous operation and integration of chemical processes within aqueous filled micro-compartments is providing opportunities for the development of programmable microscale materials with cell-like functions, dynamics and collective behaviors. Membrane-bounded synthetic protocells can be produced in the form of lipid vesicles^1–3^, polymersomes^4–7^, polypeptide capsules^8,9^, dendrimersomes^10,11^, inorganic colloidosomes^12–14^, semi-permeable protein–polymer microcapsules (proteinosomes)^15–18^, and semi-permeable bio-inorganic microcapsules^19^. In addition, coacervate micro-droplets are being exploited as membrane-free protocell models as they selectively sequester key functional components such as biomolecular substrates, proteins, polynucleotides, ribozymes, ribosomes, and chloroplasts^20–23^, and support photochemical reactions, enzyme/ribozyme cascades and gene expression within their molecularly crowded interior^24–28^. As protocell models, coacervate droplets can be stabilized by enclosure within fatty acid or block copolymer membranes^29,30^, endocytosed within liposomes^31^ and proteinosomes^32,33^, enlisted as predatory protocells^34^, exploited as functional modules in prototissues^35^ and sensing platforms^36^, and reconfigured into fatty acid vesicles^32,37^ or membrane-bounded coacervate vesicles^38^. In the latter case, coacervate droplets prepared from polydiallydimethylammonium chloride (PDDA) and adenosine 5′-triphosphate (ATP) were transformed by electrostatically induced complexation of a polyoxometalate (POM) (sodium phosphotungstate, [PW_11_O_39_]^7−^; PTA) with PDDA molecules present at the droplet surface. The concomitant changes in osmotic pressure gave rise to a complex three-tiered microstructure comprising a semi-permeable negatively charged PTA/PDDA outer membrane, a sub-membrane coacervate shell containing guest components, and an internal aqueous lumen^38^. Although the resulting POM coacervate vesicles (PCVs) exhibited no intrinsic chemical reactivity, they could be rendered functional by encapsulation of enzymes within the coacervate sub-membrane layer.

In this paper, we extend the above approach to the spontaneous preparation of catalytic PCVs by transforming the PDDA/ATP coacervate droplets in the presence of mixture of PTA and a bio-inspired Ru(IV)-based POM polyanionic catalyst (Na_10_[Ru_4_(μ–O)_4_(μ–OH)_2_(H_2_O)_4_(γ–SiW_10_O_36_)_2_]; Ru_4_POM)^39,40^ to produce synzyme active protocells (Ru_4_PCVs) with catalase-like membrane activity. We use Ru_4_POM as an exogenous agent as it is readily synthesized, chemically stable, and forms a strong electrostatic complex with PDDA polycations present at the droplet surface due to its high negative charge. Moreover, the adamantane-like Ru-oxo core of the synzyme comprises four redox active sites connected through μ-oxo and μ-hydroxo bridges, which mimic the natural oxygen evolving photosynthetic center to produce an effective bio-inspired oxygenic catalyst^41,42^. We assess the synzyme activity of the membrane-integrated Ru_4_POM structural unit by determining the levels of O_2_ produced when populations of the Ru_4_PCVs are incubated with aqueous H_2_O_2_, and exploit the collective catalase-like activity to design a multi-compartmentalized protocell model capable of endogenously driven buoyancy. By incorporating competitive synzyme and peroxidase reaction pathways within individual protocells we prepare spatially organized networks of Ru_4_PCVs that undergo parallel catalytic processing. Alternatively, we use the same competitive reactions to implement a spatially distributed signaling pathway within a ternary protocell community dispersed in aqueous medium or encapsulated within water-in-oil emulsion droplets. In both cases, the consortium consists of a transmitter population of synzyme-inactive glucose oxidase (GOx)-containing PCVs that endogenously produce a H_2_O_2_ signal and two competitive receiver populations of peroxidase-active or synzyme-active PCVs and Ru_4_PCVs, respectively. Taken together, our results provide opportunities for the fabrication of new types of catalytic micro-compartments based on membrane-integrated POM clusters and provide a step towards the development of protocell reaction networks.

## Results

### Catalytic activity of synzyme protocells

**Fig. 1:**
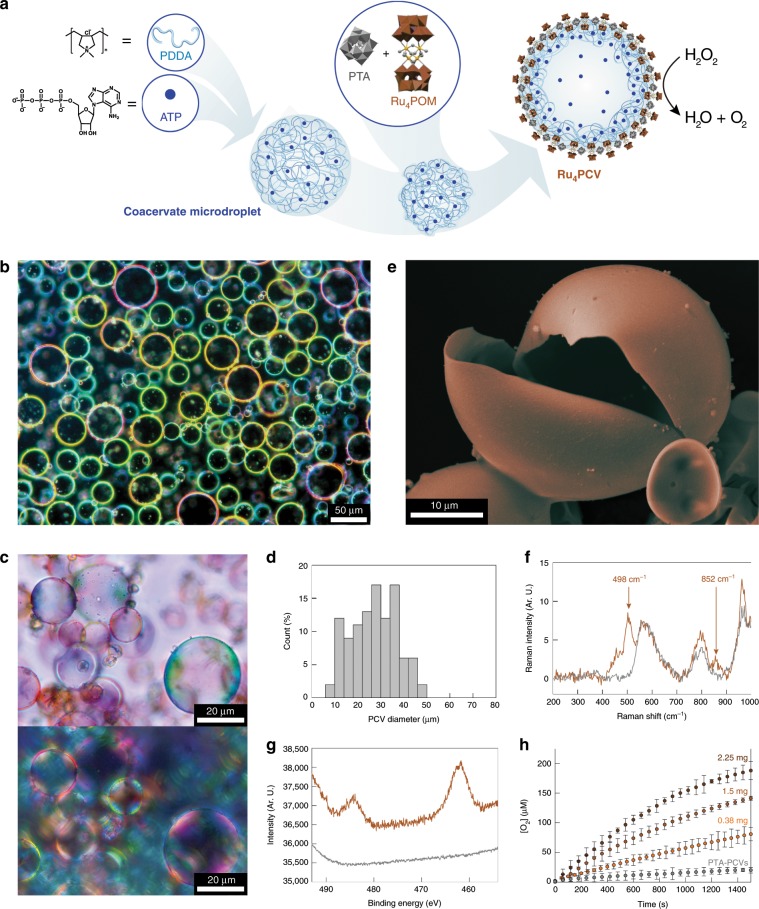
Self-assembly and catalytic activity of synzyme protocells. **a** Scheme showing the self-assembly of catalytic Ru_4_PCVs. Membrane-free non-catalytic PDDA/ATP coacervate micro-droplets are reconfigured into catalytic membrane-bounded coacervate vesicles by electrostatically induced surface binding of Ru_4_POM and PTA polyanions. Membrane formation is accompanied by changes in osmotic pressure that generate a three-tiered micro-architecture consisting of a Ru_4_POM/PTA/PDDA catalytic membrane, a PDDA/ATP sub-membrane coacervate shell, and an expanded aqueous lumen. **b** Dark-field microscopy image showing a population of Ru_4_PCVs in aqueous solution. **c** Bright-field microscopy image of a lyophilized sample of Ru_4_PCVs (top), and corresponding cross-polarized image (bottom) showing birefringence. **d** Size distribution for Ru_4_PCVs produced by addition of a Ru_4_POM/PTA mixture (7:1 molar ratio) to coacervate microdroplets after 30 s of stirring. **e** SEM image of a lyophilized single Ru_4_PCV showing hollow interior and smooth outer membrane surface. Images have been artificially colored to mimic the real tinge of the material. **f** Raman spectra of lyophilized Ru_4_PCVs (light brown) and PTA-CVs (gray) powders. Absorbance bands: 498 cm^−1^ (Ru–O–Ru *str*, Ru_4_(μ–O)_4_); 852 cm^−1^ (Si–O *str*, [γ-SiW_10_O_36_]^8−^); 575 cm^−1^ (O–P–O *def*); 798 cm^−1^ (W–O–W *def*); 978 cm^−1^ (W=O *str*)^40,52,53^. **g** XPS spectra (Ru 3p region) of lyophilized Ru_4_PCVs (light brown) and PTA-CVs (gray) powders. Photoemission lines for Ru(IV) are observed at 484.5 eV (3p_1/2_) and 462.5 eV (3p_3/2_)^54,55^ in the Ru_4_PCV sample. **h** Room temperature kinetic plots of dioxygen evolution against Ru_4_PCVs concentration (mg in PBS buffer (500 μL, 10 mM, pH 6.5, Ar purged)) at [H_2_O_2_] = 2.8 M showing protocell-mediated synzyme activity. Absence of activity for the PTA-CVs control samples is also shown. Error bars: standard deviation.

Membrane-free PDDA/ATP coacervate micro-droplets were structurally and compositionally reconfigured into membrane-bounded coacervate vesicles by addition of an aqueous solution of Ru_4_POM and PTA polyanions (Fig. 1a). Typically, Ru_4_PCVs prepared at a PTA: Ru_4_POM molar ratio of 7:1 were intact, non-aggregated, birefringent, and polydisperse (mean diameter, 25 ± 15 µm; 30 s stirring time) (Fig. 1b–d and Supplementary Fig. 1). SEM images confirmed a hollow interior and smooth pliant outer membrane, 500–800 nm in thickness (Fig. 1e and Supplementary Fig. 2), which was consistent with a three-tiered microstructure as described previously for PTA-CVs^38^. Ru_4_PCVs with larger sizes and increased polydispersity were obtained by increasing the extent of coacervate droplet coalescence prior to reconfiguration (Supplementary Fig. 3). Ru_4_PCVs were also prepared at lower PTA: Ru_4_POM molar ratios, but were prone to osmotic collapse at Ru_4_POM levels greater than 12 mol%. Zeta-potential measurements showed a marked decrease in surface potential for the PDDA/ATP droplets from *ca*. 0 mV to −35.6 ± 1.4 mV after formation of the Ru_4_PCVs (Supplementary Fig. 4), which was attributed to complexation of excess POM polyanions with PDDA at the droplet/water interface. No change in the surface charge of the Ru_4_PCVs was observed when the Ru_4_POM concentration used in the PCV preparation was varied from 0.12 to 0.48 mmol. We attributed this to the large molar excess of POMs required to trigger the coacervate-to-vesicle reconfiguration and the higher negative charge of Ru_4_POM compared to PTA. FT-IR, Raman and XPS spectra confirmed the presence of structurally intact PTA and Ru_4_POM species within the PCV membrane (Fig. 1f, g and Supplementary Figs. 5, 6). Quantitative analysis of the XPS data gave a W: Ru atomic ratio of 10 ± 4, equivalent to an average PTA:Ru_4_POM molar ratio of 1.7:1 in the PCV membrane (Supplementary Fig. 6). As the starting ratio was 7:1, the PDDA/POM membrane was considerably enriched in Ru_4_POM, consistent with the increased electrostatic interactions associated with the higher negative charge.

Given these observations, we tested the in situ synzyme activity of the Ru_4_POM structural unit when integrated into the membrane of the artificial protocells. Specifically, we determined the catalase-like activity by incubating populations of the Ru_4_PCVs with H_2_O_2_, followed by measurement of the O_2_ produced over a period of 60 min (Fig. 1h and Supplementary Fig. 7). The Ru_4_PCVs remained intact under the experimental conditions employed (Supplementary Fig. 8). The kinetic plots indicated that the rate of O_2_ production was proportional to the number of Ru_4_PCVs with a second order rate constant of 43.0 × 10^−3^ M^−1^ s^−1^ (5.90 × 10^−8^ mg^−1^ s^−1^). The rate constant was three orders of magnitude smaller than that determined for homogeneous catalysis in aqueous solutions of Ru_4_POM (36.8 M^−1^ s^−1^)^43^, which was attributed to attenuated diffusion of H_2_O_2_ within the POM/polyelectrolyte matrix of the PCV membrane. However, the measured rate constant was comparable to values determined previously for Ru_4_POM in heterogenous catalysis^43,44^, suggesting that the catalytic activity was not unduly hindered within the protocell membrane. In contrast, no catalytic activity was observed when populations of the ruthenium-free PTA-CVs were exposed to H_2_O_2_ under identical reaction conditions.

**Fig. 2:**
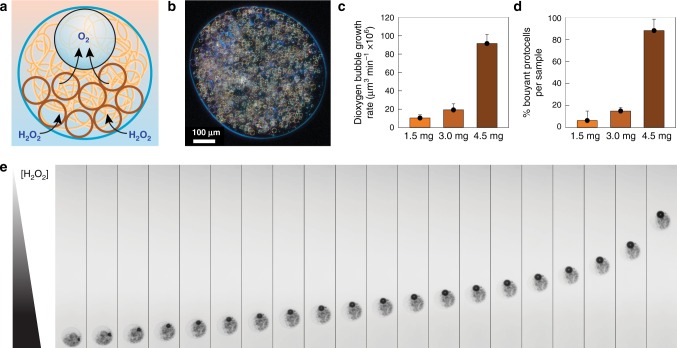
Collective synzyme activity in multi-compartmentalized protocells. **a** Scheme illustrating the design and operation of a multi-compartmentalized model protocell exhibiting proto-organelle-mediated buoyancy. The micro-architecture is suspended in water and consists of a semi-permeable aminoclay/DNA outer membrane (blue circle) that encages a viscous aqueous solution of DNA (orange curved line) containing tens of thousands of Ru_4_PCVs (brown circles). Addition of H_2_O_2_ results in proto-organelle-mediated dioxygen production, gas bubble growth and buoyancy-induced vertical movement of the host–guest ensemble. **b** Dark field microscopy image recorded in water showing a single aminoclay/DNA protocell containing a dense population of catalytically active Ru_4_PCVs proto-organelles. Typical mean sizes of the host aminoclay/DNA microcapsule and guest Ru_4_PCVs were 530 ± 13 μm and 25 ± 15 µm, respectively; estimated free internal volume, 50 ± 10%; estimated number of encapsulated Ru_4_PCVs, 20 ± 10 × 10^3^. **c** Plot showing rate of dioxygen bubble formation (μm^3^ min^−1^) against Ru_4_PCV loading in aminoclay/DNA protocells after exposure to H_2_O_2_ (4.9 M). Loadings are given as mg of Ru_4_PCVs used to prepare the PCV/DNA dispersion. Error bars: standard deviation. **d** Plot showing the percentage of buoyant multi-compartmentalized protocells against Ru_4_PCV loading after exposure to H_2_O_2_ (4.9 M). Error bars: standard deviation. **e** Composite image (see Supplementary Movie 1) showing onset and progression of buoyancy for a single Ru_4_PCV-containing aminoclay/DNA protocell initially located at the bottom of a water-filled glass cuvette containing a gradient of H_2_O_2_ (higher concentration towards the bottom). The oxygen bubble is observed as a highly refractive black spot inside the protocell. The population of encapsulated Ru_4_PCVs is observed as dark material within the aminoclay/DNA micro-capsules. Frames displayed at intervals of 1.2 s.

Having established the synzyme activity of the individual Ru_4_PCVs, we exploited their collective catalase-like activity in a multi-compartmentalized protocell model prepared by encapsulation of tens of thousands of multiple Ru_4_PCVs within individual semi-permeable aminoclay/DNA microcapsules typically 500 μm in size (Fig. 2a). Entrapment of the Ru_4_PCVs within the microcapsules produced a dense population of artificial proto-organelles capable of generating dioxygen in the presence of exogeneous H_2_O_2_ (Fig. 2b and Supplementary Fig. 9). Increasing the number of encapsulated Ru_4_PCVs above a critical level resulted in the growth of predominantly single gas bubbles within the multi-compartmentalized protocells (Fig. 2c). As a consequence, an increasing percentage of the nested micro-architectures became buoyant (Fig. 2d), resulting in vertical motility (Fig. 2e and Supplementary Movie 1). Typically, the protocells migrated with an acceleration of 7.5 ± 0.1 μm s^−2^ when placed at the bottom of a water column containing a gradient of H_2_O_2_ (Supplementary Fig. 10). As expected, multi-compartmentalized protocells containing ruthenium-free non-catalytic PTA-CVs guests were not triggered by H_2_O_2_ and remained immobile in the presence of the chemical stimulus (Supplementary Fig. 11 and Supplementary Movie 1).

### Parallel catalytic processing in synzyme protocells

**Fig. 3:**
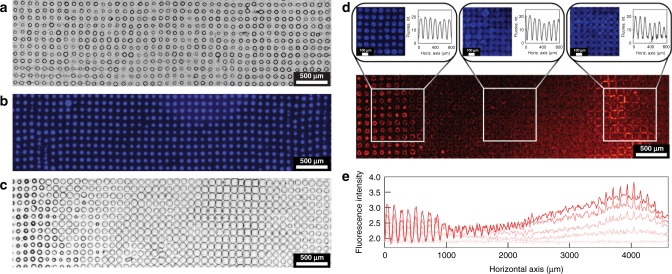
Parallel catalytic processing in synzyme protocells. **a** Brightfield microscopy image showing an acoustically trapped 2D array of immobilized HRP-containing PDDA/ATP coacervate microdroplets. The field of view is 5.0 × 1.5 mm. **b** Corresponding fluorescence microscopy image showing the homogenous distribution of sequestered Dylight405-labeled HRP (blue fluorescence) within individual coacervate droplets throughout the acoustically patterned array. **c** Brightfield microscopy image showing reconfiguration of a uniform population of HRP-containing coacervate droplets into a spatially separated trimodal community after exposure to a unidirectional PTA/Ru_4_POM chemical gradient. The PTA/Ru_4_POM mixture (7.5 µL, PTA: Ru_4_POM = 7:1 mol/mol; [PTA] = 17.6 mM; [Ru_4_POM] = 2.4 mM; pH 6.5) was injected to the left of the image at a distance of 15 mm from the observation window (5.0 × 1.5 mm). Distinct zones of non-contact small-sized Ru_4_PCVs (left side), medium-sized Ru_4_PCVs with membrane-membrane contacts (center), and highly expanded Ru_4_PCVs showing compression-induced deformation (center right) are observed (see Supplementary Movie 2). **d** Fluorescence microscopy images of an array of HRP-containing Ru_4_PCVs 16 min after homogeneous addition of H_2_O_2_ and Amplex Red (final concentrations 40 and 10 μM, respectively). The bottom image shows zones (left and right sides) of red fluorescence arising from protocells operating with a dominant HRP-mediated pathway giving rise to a resorufin output. The central region shows only background levels of red fluorescence due to prevailing synzyme activity and competitive decomposition of H_2_O_2_ to O_2_ and water_._ A homogenous distribution of coacervate-sequestered Dylight405-labeled HRP (blue fluorescence) is observed throughout the array (top images). Selected regions are delineated by the white squares and top images show blue fluorescence images and corresponding fluorescence intensity line profiles. **e** Time-dependent averaged resorufin fluorescence line intensity profiles recorded across the array shown in **d**. Increasing color saturation indicates increased elapsed time. Time intervals = 0, 4, 8, 12, and 16 min.

Competitive synzyme and enzyme reaction pathways were established within individual Ru_4_PCVs by preparing multi-catalytic protocells capable of parallel reaction processing. For this, horseradish peroxidase (HRP) was sequestered within the coacervate micro-droplets prior to structural reconfiguration to produce Ru_4_PCVs that when exposed to aqueous H_2_O_2_ displayed simultaneous membrane-mediated O_2_ production (Ru_4_POM activity) and endogenous peroxidation of membrane-permeable organic substrates (HRP activity). We investigated this system using spatially organized arrays comprising hundreds of immobilized HRP-containing Ru_4_PCVs produced by acoustic standing wave micro-droplet patterning^45^. Initially, a defect-free 2D array of uniform HRP-containing PDDA/ATP coacervate micro-droplets was generated in situ at the nodes of an acoustic pressure field produced within a custom-made acoustic trapping chamber (Fig. 3a). Fluorescence microscopy images showed a homogenous distribution of sequestered Dylight405-labeled HRP (Fig. 3b), indicating that the potential for endogenous peroxidase activity was uniform throughout the microdroplet community. We then established systematic differences in the catalase-like activity across the array by reconfiguring the spatially organized HRP-containing coacervate micro-droplets using a unidirectional POM chemical gradient. This was achieved by carefully injecting a PTA/Ru_4_POM (molar ratio = 7:1) solution from one side of the trapping device such that the membrane-restructuring agents slowly diffused through the chamber and progressively triggered the coacervate-to-vesicle transformation along an advancing reaction-diffusion planar wave front^46^. As a consequence, the uniform community of immobilized coacervate droplets transformed into a ternary population consisting of three spatially separated zones of birefringent Ru_4_PCVs that were morphologically graded along the diffusion direction (Fig. 3c, Supplementary Fig. 12, and Supplementary Movie 2). The differentiated array comprised Ru_4_PCVs that were (i) contracted in volume and spatially discrete (mean diameter, 77 ± 6 μm; membrane-to-membrane separation, 38 ± 7 μm); (ii) slightly expanded in volume (mean diameter, 108 ± 8 μm) with membrane-membrane contacts; or (iii) highly expanded in volume (mean diameter, 114 ± 8 μm) and therefore deformed by membrane-membrane compression forces. Formation of the contracted protocells occurred in regions closest to the point of injection (high PTA and Ru_4_POM levels), suggesting that the collapsed morphology was due to assembly of the semi-permeable membrane under hypertonic (high ionic strength) conditions. In contrast, the two types of swollen Ru_4_PCVs were positioned in regions further away from the injection point where the POM concentrations were decreased (hypotonicity) due to successive binding of PTA/Ru_4_POM anions to the coacervate droplets during transit of the reaction-diffusion gradient. Under these conditions, the protocells swell due to ATP-mediated osmotically induced ingress of water^38^.

Having established a morphologically graded community of immobilized HRP-containing Ru_4_PCVs, we homogeneously exposed the differentiated populations to a mixture of H_2_O_2_ and Amplex Red and monitored the HRP-mediated generation of the red fluorescent product resorufin as a proxy for the degree of competition between the endogenous peroxidase and membrane-mediated catalase-like activities of the multi-catalytic protocells. Ru_4_PCVs without HRP were inactive with regard to Amplex Red peroxidation, indicating minimal levels of crosstalk between the parallel catalytic pathways (Supplementary Fig. 13). Interestingly, although the HRP content was similar for all Ru_4_PCVs in the array, the competition between the enzyme and synzyme activities within individual protocells was spatiotemporally determined. For example, when the transitions between the differentiated protocell populations were engineered to occur within the central observation window of the device chamber (i.e., 15 mm from the point of injection), a high level of resorufin production was observed in the sub-membrane coacervate layer of the small contracted Ru_4_PCVs (Fig. 3d). This was consistent with dominant HRP activity and a reduced level of Ru_4_POM in the membrane of the reconfigured protocells produced in this region of the device. As preferential binding of Ru_4_POM to the coacervate droplets was observed under equilibrium conditions, we attributed the decrease in catalase-like activity to selective depletion of the synzyme as the PTA/Ru_4_POM chemical gradient advanced towards the observation window. In contrast, the slightly expanded Ru_4_PCVs produced adjacent to the region of HRP-dominant contracted Ru_4_PCVs showed negligible red fluorescence (Fig. 3d, e), indicating prevailing catalase-like activity and down-regulation of resorufin production in the parallel catalytic pathways. Interestingly, the exchange in chemical processing was observed downstream for thirty or so rows of protocells after which the reactivity reverted to predominant resorufin production in the highly expanded Ru_4_PCVs (Fig. 3d, e). We attribute these observations to localized changes in membrane composition that originate from fluctuations in the PTA: Ru_4_POM molar ratio as the reaction-diffusion gradient migrates unidirectionally through the protocell array. Initially, the PTA:Ru_4_POM ratio increases above a value of 7:1 because Ru_4_POM is preferentially depleted compared with PTA. The resulting higher levels of PTA then lead to more competitive PTA binding, which in turn lowers the PTA:Ru_4_POM ratio at the advancing diffusion front to generate oscillations in the composition of the PCV membrane. As a consequence, both the contracted and highly expanded morphological forms although spatially separated as populations display dominant HRP activity (high PTA:Ru_4_POM ratios), whilst the slightly expanded protocells that divide these two zones show higher synzyme activity (lower PTA:Ru_4_POM ratios). In contrast, no spatiotemporal catalytic response was observed in arrays of HRP-containing ruthenium-free PTA-CVs exposed to H_2_O_2_ and Amplex Red (Supplementary Fig. 14).

### Chemical signaling in multi-catalytic protocell communities

**Fig. 4:**
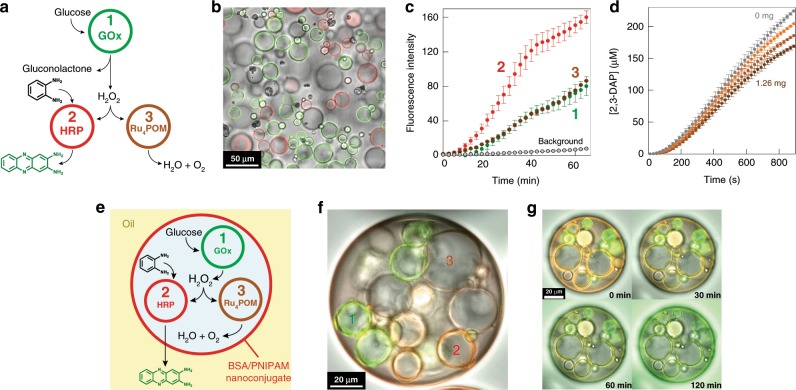
Chemical signaling in distributed and compartmentalized multi-catalytic protocell communities. **a** Scheme showing a ternary population comprising a single transmitter protocell (FITC-tagged GOx-containing PTA-CVs; green circle, 1) and two competitive receiver protocells (RITC-tagged HRP-containing PTA-CVs (red circle, 2) and untagged Ru_4_PCVs (brown circle, 3). A glucose input generates a protocell-mediated H_2_O_2_ signal that in the presence of o-PD activates peroxidase and synzyme activities simultaneously to give two competitive outputs (green fluorescent 2,3-DAP or O_2_, respectively). **b** Superimposed brightfield and fluorescence confocal microscopy image showing aqueous mixture of 1 (green fluorescence), 2 (red fluorescence) and 3 (no fluorescence) in PBS buffer (0.01 M, pH 6.5) prior to addition of glucose. Relative numbers of protocells was 1:1:1. **c** Plots of time-dependent changes in green fluorescence intensity associated with 2,3-DAP production and transfer in different individual protocells shown in **b** after addition of glucose (final concentration, 50 mM) (see Supplementary Movie 3). On receipt of the H_2_O_2_ signal, production of 2,3-DAP occurs in 2 (red data points) followed by equal rates of diffusive transfer and capture of 2,3-DAP by protocells 1 (green) and 3 (brown). Minimal background green fluorescence is observed (gray plot). Error bars: standard deviation. **d** Graph showing the time-dependent generation of 2,3-DAP as determined by plate reader measurements on dispersed ternary communities comprising constant numbers of 1 (0.08 mg) and 2 (0.08 mg), and increasing numbers of 3 (Ru_4_PCVs; 0 (gray plot), 0.23 (light brown), 0.63 (brown), and 1.26 mg (dark brown). Reactions were initiated by addition of glucose and o-PD at final concentrations of 50 mM and 1 mM, respectively. Increased numbers of Ru_4_PCVs progressively decrease the production of 2,3-DAP. Error bars: standard deviation. **e** Scheme showing compartmentalization of a ternary proto-organelle population capable of operating a parallel signaling cascade within individual water-in-oil emulsion droplet. The emulsion droplets are stabilized by a self-assembled monolayer membrane of a protein-polymer (BSA/PNIPAM) nanoconjugate and serve as a host for the synthetic proto-organelles. **f** Superimposed brightfield and fluorescence confocal microscopy image of a single water-in-oil emulsion droplet containing an aqueous mixture of protocells 1 (green fluorescence), 2 (red fluorescence) and 3 (no fluorescence). **g** As shown in **f** but at various reaction times (*t* = 0, 30, 60, and 120 min) and in the presence of glucose and o-PD in the water phase (50 and 1 mM, respectively). The video time-shots show a progressive increase in green fluorescence within protocells 1, 2, and 3 as well as in the oil phase due to 2,3-DAP production in 2 (see Supplementary Movie 4).

As an alternative approach to implementing synzyme/enzyme parallel processing within individual protocells, we spatially distributed the two functionalities by preparing separate populations of RITC-labeled HRP-containing PTA-CVs and unlabeled Ru_4_PCVs. After mixing the two populations, we added a population of FITC-tagged GOx-containing PTA-CVs to produce a ternary community capable of chemical communication and signaling (Fig. 4a, b). In general, addition of glucose in the presence of the HRP-substrate *o*-phenylenediamine (*o*-PD) produced an endogenous H_2_O_2_ signal that was diffusively transmitted from the GOx-containing PTA-CVs to the two types of receiver protocells, which catalytically processed the signal in parallel. To monitor the peroxidation reaction against catalase-like activity we used fluorescence microscopy to detect the production and trafficking of the green fluorescent peroxidation product 2,3-diaminophenazine (2,3-DAP) within and between the different individual protocells (Fig. 4c). Oxygen production specifically within the Ru_4_PCVs was not monitored due to experimental constraints. As expected, production of 2,3-DAP occurred specifically within the HRP-containing PTA-CVs, where it was initially sequestered to give an intense green fluorescence within the membrane and sub-membrane layer of the PCVs (Supplementary Movie 3). This was followed by slow efflux of the fluorescent product and subsequent sequestration of 2,3-DAP by both the GOx-containing PTA-CVs and Ru_4_PCVs (typically within 20 min). Minimal levels of green fluorescence were recorded in the aqueous background (Fig. 4c), indicating that *ca*. 98% of the peroxidation product was retained within the protocell community. Increasing the number of Ru_4_PCVs included in ternary populations prepared at a constant GOx-PTA-CV:HRP-PTA-CV ratio (1:1 wt/wt) and fixed substrate concentrations resulted in a corresponding decrease in 2,3-DAP production due to enhanced synzyme activity (Fig. 4d and Supplementary Fig. 15). In general, Ru_4_PCV: PTA-CV number ratios greater than 3.0 were required to attenuate the peroxidase activity, indicating that parallel processing of the endogenous H_2_O_2_ signal was less pronounced than compared with integration of the catalytic pathways into individual protocells (Fig. 4d).

Finally, we adopted the above model system as a step towards a nested synthetic protocell capable of operating an endogenous sequence of multi-catalytic inter-proto-organelle pathways. For this, we incarcerated the above ternary population along with glucose and *o*-PD within individual water-in-oil emulsion droplets enclosed within a protein-polymer nanoconjugate membrane (Fig. 4e, f and Supplementary Fig. 16)^15^ Production of 2,3-DAP in the compartmentalized community was monitored by optical and fluorescence microscopy (Fig. 4g and Supplementary Movie 4). The images revealed a sequence of spatiotemporal responses that were similar to that observed in communities of the spatially distributed protocells except that 2,3-DAP was progressively removed from the reaction environment by solubilization in the oil phase.

## Discussion

In summary, membrane-free molecularly crowded PDDA/ATP coacervate microdroplets are spontaneously reconfigured into catalytic membrane-bounded coacervate vesicles in the presence of Ru_4_POM and PTA polyanionic clusters. Catalytic dismutation of H_2_O_2_ at the tetra-Ru(IV) synzyme core is associated specifically with the membrane of the coacervate vesicle and can be used collectively within large multi-compartmentalized synthetic protocells to internally generate sufficient amounts of oxygen that give rise to catalytically powered gas-induced flotation. Incorporation of membrane-mediated synzyme and endogenous peroxidase reaction pathways within individual protocells provides a step to competitive parallel catalytic processing, which can be expressed collectively in graded communities of spatially organized Ru_4_PCVs. The two catalytic pathways can also be spatially separated by incorporation into individual populations of peroxidase-active or synzyme-active PCVs and Ru_4_PCVs, respectively. When used in conjunction with a H_2_O_2_ signaling population of GOx-active PCVs, this gives rise to a distributed transmitter-receiver network in a ternary protocell community, which can be encapsulated and subsequently exploited to implement synzyme/enzyme parallel processing within a nested protocell model.

Taken together, our results open up the possibility of using inorganic POM catalysts for the spontaneous self-assembly of membrane-bounded micro-compartments with bioinspired properties. As the synzyme protocells are assembled via a single-step aqueous-based process, the methodology could offer potential advantages in terms of simplification and scale-up compared with Pickering emulsification (colloidosomes, proteinosomes, and emulsion droplets) and microfluidic processing (lipid vesicles, hydrogel particles). Moreover, the ability to spontaneously generate hierarchical Ru_4_PCVs in water could offer numerous functional advantages over vesicles, polymersomes and hydrogel particles, which generally exhibit homogeneous structures and compositions. For example, vis-à-vis the significant potential of tenable POM structures for bio-inspired catalysis^47–50^, the ability to synthesize such compounds in relatively large quantities, and the resilience of POMs under adverse reaction conditions compared to biomolecular catalysts, it seems feasible that communities of synzyme protocells could provide a step towards synthetic metabolic networks based on light-activated stimuli^47^. More generally, the development of microscale catalytic materials with programmable cell-like functions, dynamics and interactivity could provide new opportunities in microscale systems chemistry, soft matter bioengineering, and synthetic protobiology.

## Methods

### Synthesis of Ru_4_POM

Na_10_[Ru_4_(μ–O)_4_(μ–OH)_2_(H_2_O)_4_(γ–SiW_10_O_36_)_2_] (Ru_4_POM) was synthesized according to the procedure established by Sartorel and co-workers^40^. In brief, in a typical synthesis, 188 mg (0.72 mmol) of ruthenium chloride trihydrate were dissolved in 30 mL of deionized water. To this solution, 1 g (0.336 mmol) of K_8_[γ-SiW_10_O_36_]·12H_2_O was directly added. The resulting dark brown solution was kept at 70 °C for 1 h. After the thermal treatment, the pH dropped to 1.8, and the solution was filtered. An excess of CsCl (4.4 g, 26 mmol) was then added to precipitate the product as the corresponding cesium salt, which was then washed three times with 2–3 mL of cold water. Nine hundred and eighty milligram of crude Ru_4_POM cesium salt were obtained in 85% yield. The cesium salt was then dissolved in 100 mL of water, and the corresponding sodium salt was obtained by passing it through a Na^+^ exchange resin (Amberlite IR120 sodium form). Eight hundred and fifty milligram of crude Ru_4_POM sodium salt were obtained. The crude sodium salt was then purified by size-exclusion chromatography (Sephadex G-50 column, Sigma-Aldrich). The solvent was finally removed to give 700 mg of purified Ru_4_POM sodium salt in 70% yield (based on initial tungsten).

### Preparation of Ru_4_PCVs

In sequence, PDDA (10 mM, 500 µL, pH 6.5) and ATP stock solutions (10 mM, 500 µL, pH 6.5) were added to a 1.75 mL vial and stirred (1700 rpm, 30 s.) to form PDDA/ATP coacervate microdroplets (PDDA_monomer_:ATP molar ratio = 1:1). To this stirring solution a freshly prepared mixture of PTA and Ru_4_POM solution (100 µL), prepared by mixing a stock solution of PTA (80 µL, 22 mM, pH 6.5) and a stock solution of Ru_4_POM (20 μL, 12 mM, pH 6.5), was quickly injected. The PCVs dispersion was stirred for 30 s at 1700 rpm and then transferred into an Eppendorf tube. The PCVs were let to sediment for 30 min, subsequently the supernatant was carefully removed and replaced with 500 μL of PBS buffer 0.01 M pH 6.5. This washing procedure was repeated three times to finally give a batch of Ru_4_PCVs dispersed in 500 μL of PBS buffer (10 mM, pH 6.5). Each batch of Ru_4_PCVs prepared by this methodology contained 1.5 ± 0.5 mg of PCVs as determined by lyophilizing and weighing the samples prepared as described above but washed with MilliQ water instead of PBS buffer. Alternatively, gentle centrifugation (2 min, 94×*g*) was used to speed up the washing process; however, small PCV aggregates were often produced by this method.

Before each experiment, the Ru_4_PCV samples were resuspended by stirring on a vortex to obtain a homogenous PCV dispersion.

### Preparation of PTA-CVs

PTA-CVs were produced using the procedure described above, with the exception that only the PTA stock solution (100 µL, 22 mM, pH 6.5) was added to the stirred coacervate phase instead of the Ru_4_POM/PTA mixture.

### Synthesis of aminoclay

Aminopropyl-functionalized magnesium phyllosilicate (AMP; amino-clay) was prepared according to the procedure established by Burkett and co-workers^51^. In a typical synthesis, AMP clay was prepared by dropwise addition of 3-aminopropyltriethoxysilane (1.3 mL, 5.85 mmol) to an ethanol solution of magnesium chloride (0.84 g, 3.62 mmol). The aminoclay precipitated within 5 min as white powder, which was stirred overnight, before being collected by centrifugation, washed with ethanol for three times and dried at 40 °C.

### Ru_4_PCV-containing aminoclay/DNA synthetic protocells

First, three unwashed samples with different amount of Ru_4_PCVs (1.5, 3.0, and 4.5 mg) were prepared according to the procedure described above and allowed to sediment for 30 min. In general, the supernatant was then removed, and the PCV sediment was mixed with 200 µL of aqueous DNA (20 mg mL^−1^). The resulting homogeneous stable dispersion was then diluted to 500 µL with RNA-free water and transferred to a 1 mL plastic syringe equipped with a hypodermic needle (25 gauge) and a nozzle. To generate Ru_4_PCV-containing aminoclay/DNA synthetic protocells, the homogeneous DNA/PCV dispersion was extruded at a flow rates of 10 µL min^−1^. A flow of air (2 L min^−1^) was directed through a Tefzel tube (0.50 mm internal diameter, VICI, JR-T-082-M3) into the nozzle to generate a coaxial air jet around the hypodermic needle to shear the extruded DNA/PCV dispersion into micro-droplets into a freshly exfoliated aminoclay solution (5 mg mL^−1^) placed below the nozzle. The microcapsules were washed with dilute dispersion of the aminoclay solution (0.5 mg mL^−1^) and stored in the same solution.

PTA-CV-containing aminoclay/DNA microcapsules for control experiments were prepared following the same procedure but using an unwashed 3-batch-concentrated PTA-CV dispersion in place of Ru_4_PCVs.

### Preparation of 2D PCV arrays

The PDDA/ATP coacervate micro-droplet array was prepared in a custom-built acoustic trapping device with a square arrangement of four piezoelectric transducers (Noliac, NCE 51, L15 × W2 × T1 mm). The opposing transducer pairs were wired in parallel, driven by two signal generators (Agilent 33220a-001), and each connected to an oscilloscope (Agilent DSOX2014A). A PEGylated glass coverslip was attached with adhesive to the bottom of the device. Neutrally charged PDDA/ATP coacervate micro-droplets were prepared in situ by adding an aqueous solution of ATP (100 μL, 50 mM, pH 6.5) to an aqueous solution of PDDA (1 mL, 5 mM monomer, 100–200 kDa, pH 6.5) in the presence of the two orthogonal acoustic standing waves generated from opposing transducer pairs operating at 6.76/6.78 MHz (10 V). The mixtures were stirred to ensure homogeneous formation of the coacervate droplets in the square chamber. Continuous coalescence of the trapped coacervate droplets at the pressure nodes (30 min), produced a micro-droplet array. The supernatant in the acoustic chamber was carefully removed and exchanged with Milli-Q water for three times under the same acoustic force field. Then, the acoustic field was switched off, and a mixture of PTA and Ru_4_POM (7.5 µL, PTA/Ru_4_POM 7:1 mol/mol; [PTA] = 17.6 mM; [Ru_4_POM] = 2.4 mM; pH 6.5) was added to the acoustic trapping chamber from one side of the device. Optical microscope images were recorded at the center of the trapping chamber with an observation window of 5 × 1.5 mm.

For the control experiments we adopted the same procedure described above, but instead of a mixture of PTA and Ru_4_POM we added a solution of PTA (7.5 µL, 20 mM) to the acoustic trapping chamber.

### Preparation of FITC-tagged GOx and RITC-tagged HRP

Enzymes GOx or HRP (20 mg) were dissolved in Na_2_CO_3_ buffer (7 mL, 0.1 M, pH 8.5). Solutions of RITC or FITC in DMSO (1 mg mL^−1^) were freshly prepared. The RITC solution (200 µL) was added dropwise to the stirring HRP solution, and the FITC solution (200 µL) was added dropwise to the stirring GOx solution. The solutions were stirred overnight at 4 °C, purified by dialysis (8 h, 2.5 L water replaced three times), and lyophilized.

The degree of labeling (DOL) of FITC-tagged GOx and RITC-tagged HRP were calculated using UV–Vis spectroscopy [FITC *ε*(495 nm) = 84,000 L mol^−1^ cm^−1^; RITC *ε*(559 nm) = 62,100 L mol^−1^ cm^−1^; GOx *ε*(280 nm) = 3,006,000 L mol^−1^ cm^−1^, HRP *ε*(403 nm) = 102,000 L mol^−1^ cm^−1^]. DOL values were determined as 58 for FITC-GOx and 3 for RITC-HRP.

### FITC-tagged GOx-containing or RITC-tagged HRP-containing PTA-CVs

FITC-tagged GOx-containing or RITC-tagged HRP-containing PCVs were prepared following our standard procedure for PTA-CVs reported in section 1.3, with the exception that the relevant solution of either FITC-tagged GOx (25 µL, 4 mg mL^−1^) or RITC-tagged HRP (25 µL, 2 mg mL^−1^) was stirred with PDDA (500 µL, 10 mM) before the addition of ATP.

To determine the partition coefficient (*K*) of FITC-tagged GOx or RITC-tagged HRP between PCV and bulk solution, the washing procedure was not performed, and images of the fluorescent PCV samples were acquired by fluorescence confocal microscopy and analysed with ImageJ software. The partition coefficient was experimentally determined as the ratio between the fluorescence intensity of the PCV PDDA/ATP submembrane and the fluorescence intensity of the bulk solution, using the following equation:

1 $$K = \frac{{F_{{\mathrm{PCV}}}}}{{F_{{\mathrm{bulk}}}}} \equiv \frac{{C_{{\mathrm{PCV}}}}}{{C_{{\mathrm{bulk}}}}},$$

where *F*_PCV_ is the fluorescence intensity of the PCV PDDA/ATP submembrane, *F*_bulk_ is the fluorescence intensity of the bulk solution, *C*_PCV_ is the concentration of enzyme in the PCV PDDA/ATP submembrane, *C*_bulk_ is the concentration of enzyme in the bulk solution.

The total concentration of enzyme in the unwashed PCV sample (*C*_Tot_; *C*_Tot_ of GOx = 88.9 µg mL^−1^; *C*_Tot_ of HRP = 44.4 µg mL^−1^) can be written as:

2 $$C_{{\mathrm{Tot}}} = C_{{\mathrm{PCV}}} + C_{{\mathrm{bulk}}}$$

This gives two unknowns and two equations, which can be solved for *C*_PCV_ and *C*_bulk_, giving:

3 $$C_{{\mathrm{PCV}}} = \frac{{KC_{{\mathrm{Tot}}}}}{{1 + K}}$$

4 $$C_{{\mathrm{bulk}}} = \frac{{C_{{\mathrm{Tot}}}}}{{1 + K}}$$

*C*_PCV_ for the FITC-tagged GOx-containing PCVs was found to be 87.0 µg mL^−1^. *C*_PCV_ for the FITC-tagged GOx-containing PCVs was found to be 42.7 µg mL^−1^.

To verify that the enzymes were effectively sequestered inside the Ru_4_PCVs and were not leaking out during the washing procedure, we put 20 μL of unwashed enzyme-filled PTACV dispersion on a microscope cover slip, further diluted it 2, 5, and 10 times, and acquired fluorescence confocal microscopy images. Using ImageJ and the method reported above we then determined *K*, which was found to increase linearly with the dilution factor (*D*) indicating that the enzymes were effectively retained within the PCV membrane.

### Synthesis of RITC-labeled BSA/PNIPAM nanoconjugate

The RITC-labeled BSA/PNIPAM nanoconjugate was prepared according to a previously established procedure^15^.

## Supplementary information

Supplementary Information

Description of Additional Supplementary Files

Supplementary Movie 1

Supplementary Movie 2

Supplementary Movie 3

Supplementary Movie 4

## Data Availability

The authors declare that all relevant data supporting the finding of this study are available within the paper and its Supplementary Information files. Additional data are available from the corresponding author upon request.
