# Supplementary material for: Catalytic processing in ruthenium-based polyoxometalate coacervate protocells
Source: Nat Commun. 2020 Jan 3;11:41. doi: 10.1038/s41467-019-13759-1 (PMC6941959; doi:10.1038/s41467-019-13759-1)
Supplement: Supplementary file 1 — Supplementary Information [file 41467_2019_13759_MOESM1_ESM.pdf]

Supplementary Information

Catalytic processing in ruthenium-based polyoxometalate coacervate protocells

Gobbo P. *et al.*

Table of Contents

1. SUPPLEMENTARY METHODS 3

1.1 MATERIALS AND METHODS 3

1.2 KINETIC STUDIES OF RU_4PCV-MEDIATED DECOMPOSITION OF H_2O_2 4

1.3 OXYGEN BUBBLE NUCLEATION IN RU_4PCV-CONTAINING AMINOCLAY/DNA SYNTHETIC PROTOCELLS 4

1.4 PARALLEL CATALYTIC PROCESSING IN SYNZYME PROTOCELLS 4

1.5 CHEMICAL COMMUNICATION WITHIN DISPERSED PTA-CV/RU_4PCV ENZYME/SYNZYME CONSORTIA 5

1.6 DETERMINATION OF PTA-CV/RU_4PCV RU_4PCVS ENZYME/SYNZYME ACTIVITIES 5

1.7 FABRICATION AND ENZYME/SYNZYME ACTIVITY OF PTA-CV/RU_4PCV-CONTAINING PROTEINOSOMES 6

2. SUPPLEMENTARY FIGURES 7

3. SUPPLEMENTARY REFERENCES 21

1. Supplementary Methods

1.1 Materials and Methods

All materials were used as received. Poly(diallyldimethylammonium chloride) (PDDA), adenosine 5′-triphosphate (ATP), phosphotungstic acid (PTA), hydrogen peroxide (H_2O_2), glucose oxidase (GOx) (252100 U mg^{-1}), horseradish peroxidase (HRP) (172 U mg^{-1}), fluorescein isothiocyanate (FITC), rhodamine B isothiocyanate (RITC), *o*-phenyldiamine (*o*-PD), ruthenium chloride trihydrate, sodium tungstate dihydrate, sodium silicate, ruthenium standard solution (1000 mg L^{-1}, in 7 wt.% HCl), tungsten standard solution (1000 mg L^{-1}, in HNO_3 and HF), and DNA sodium salt from salmon testes ($M_W \approx 1300$ kDa), magnesium chloride hexahydrate, and 3-aminopropyltriethoxysilane were purchase from Sigma-Aldrich. Sulfuric acid (H_2SO_4, 98 %, Aristar Ultra) and D-(+)-Glucose (AnalaR) were purchased from VWR. Nitric acid (HNO_3) (68 %, Fischer Primar Plus Grade) was purchased from Fischer Scientific.

Optical microscopy was performed on a Zeiss Axioskop transmitted light microscope at 4x, 10x or 20x magnification. Images were analysed with ImageJ software.

Fluorescence microscopy was performed on a Leica SP8 AOBS confocal laser scanning microscope attached to a Leica DM I6000 inverted epifluorescence microscope, at 4x, 10x or 20x magnification. Images were taken using excitation wavelengths of 405 nm (*o*-PD), 488 nm (FITC) and 561 nm (RITC) and emission wavelengths of 550-625 nm, 500-550 nm and 575-650 nm, respectively.

Raman analysis was performed on a Renishaw 2000 laser system, with samples acquired at an excitation wavelength 480 nm for 10 minutes with 60 scans. Samples of Ru_4PCVs and PTACVs were lyophilized and sputtered on a microscope slide for measurements.

ICP-AES analysis was performed on an Agilent 710 ICP-OES fitted with an Agilent SPS 3 auto-sampler. The instrument was calibrated using standard solutions of 0.2 ppm, 0.4 ppm, 0.6 ppm, 0.8 ppm and 1 ppm prepared from solution of the single element ruthenium (in 7% wt. HCl, 1000 ppm) and tungsten (in nitric acid and hydrofluoric acid, 1000 ppm). For measurements, samples of Ru_4PCVs (5.7 mg) and PTACVs (5.3 mg) were freshly prepared, lyophilized, and digested in H_2SO_4 (98 %, 1 mL) for five days. The resulting solutions were evaporated to almost dryness (heating *ca.* 4 h), and subsequently redissolved in nitric acid (1 %, 25 mL) and filtered (22 µDa particle size) before measurements. Because of the presence of the precipitate that needed to be filtered, the amounts of heavy metals might be underestimated.

X-ray photoelectron spectroscopy (XPS) was carried out using an Argus spectrometer working at a base pressure of 4.0×10^{-11} mbar. Core-level photoemission spectra were acquired in grazing incidence, *i.e.* 45° between the sample surface and the normal of the electron analyzer, with a monochromatic Al K_α (1,486.7 eV) source. The pass energy was set to 20 eV, 50 eV and 100 eV for high-resolution, survey, and high-throughput regions, respectively. The measurements were acquired at room temperature. The electron charging was neutralized by a flood gun. The binding energy scale was referenced to the carbon C-C bond in the C 1s photoemission line at 284.8 eV. Ru_4PCVs and PTACVs samples were measured as lyophilized powders.

Molecular oxygen concentration was measured using an OceanOptics NeoFox and FOSPOR-R probe with silicon overcoat. Before every measurement, the sensor was calibrated using a two-point calibration: 0 % oxygen was acquired under argon, and 20.9 % oxygen in the open air. Data was logged using NeoFox Viewer Ver. 2.40 averaging over 10 readings.

1.2 Kinetic studies of Ru_4PCV-mediated decomposition of H_2O_2

In a 1.75 mL glass vial, different amounts of Ru_4PCVs (0.38, 1.50 and 2.25 mg) were dispersed in PBS buffer (10 mM, pH 6.5) to obtain a final volume of 500 µL. Subsequently, the vial was sealed, the oxygen probe was inserted through the seal to be held above the liquid, and the PCV dispersion was stirred and purged with Ar gas. A pre-purged H_2O_2 solution (200 µL, 30 w/w %) was added using a micro-syringe and the evolution of oxygen gas was monitored for 60 min. Control experiments were conducted following the same general protocol, but either in the absence of H_2O_2 (by injecting pre-purged H_2O), or in the absence of Ru_4POM (using PTA-CVs).

The Ru_4POM-mediated decomposition of H_2O_2 follows second order kinetics[1]:

$$R = k\,[H_2O_2]\,[Ru_4POM] \qquad (1)$$

where R is the reaction rate, k is the second order rate constant, $[H_2O_2]$ is the concentration of hydrogen peroxide (2.8 M), and [Ru_4POM] is the concentration of inorganic synzyme (0.96 ± 0.02 nmol mg^{-1} of PCVs). By using this equation it was possible to calculate the catalytic response of Ru_4PCVs; typically, k = 43.0×10^{-3} M^{-1} s^{-1} (5.90×10^{-8} mg^{-1} s^{-1}) (see Supplementary Figure 7).

1.3 Oxygen bubble nucleation in Ru_4PCV-containing aminoclay/DNA synthetic protocells

Statistical studies: All experiments were performed on freshly made aminoclay/DNA protocells containing different amounts of Ru_4PCVs (1, 2 and 3 batches; see Methods *Ru_4PCV-containing aminoclay/DNA synthetic protocells*). Controlled numbers of aminoclay/DNA multicompartmentalized synthetic protocells (typically 100 µL of concentrated dispersion) were transferred into well plates using a glass pipette, and 100 µL hydrogen peroxide (30 w/w%) were added. The wells were monitored using an optical microscope, and the statistics related to the number of protocells capable of nucleating oxygen bubbles within their lumen as a function of the amount of Ru_4PCV proto-organelles was determined.

Bubble growth rate: Buoyant microcapsules were transferred using a glass pipette into a well plate containing a mixture of 100 µL of H_2O_2 and 100 µL of dilute dispersion of AMP clay (0.5 mg/mL). The growth of the microbubbles was monitored using an optical microscope and the image stacks were analysed using ImageJ software to quantify the oxygen bubble growth rate.

Protocell buoyancy experiments: Approximatively 300 µL of a dilute dispersion of aminoclay (0.5 mg/mL) were pipetted into a plastic cuvette followed by careful addition of 100 µL of hydrogen peroxide (30 w/w%) using a pipette to ensure that that the substrate and clay dispersion were preferentially located at the bottom of the cuvette. Ru_4PCV-containing aminoclay/DNA synthetic protocells or PTA-CV-containing aminoclay/DNA microcapsules were then transferred into the cuvette using a glass pipette and allowed to sink under the influence of gravity. To capture the vertical (*z* direction) motion of the protocells, a Zeiss Axioskop microscope equipped with a Canon EOS 500D camera and a 1.25x objective lens was aligned side-on to the cuvette to switch the viewing plane from *xy* to *xz*.

1.4 Parallel catalytic processing in synzyme protocells.

An aqueous solution of ATP (100 µL, 50 mM,) was added to a mixture (1 mL) of PDDA (5 mM monomer, 100–200 kDa) and horseradish peroxidase (HRP, 0.01 mg/ml) in the presence of two orthogonal acoustic standing waves (6.76/6.78 MHz, 10 V). After 30 min, the supernatant was carefully removed

and exchanged with Milli-Q water three times without disturbing the array of coacervate micro-droplets. The 2D HRP-containing PCV array was prepared by injecting a mixture of PTA and Ru_4POM (7.5 µL, PTA/Ru_4POM 7:1 mol/mol; [PTA] = 17.6 mM; [Ru_4POM] = 2.4 mM; pH 6.5) from one side of the acoustic trapping chamber. After 1 h of the injection, the supernatant in the acoustic trapping chamber was carefully removed and exchanged with Milli-Q water for three times. To avoid concentration gradients for the substrates, in an Eppendorf tube H_2O_2 (4 uL, 10 mM) and Amplex Red (1 uL, 10 mM) were diluted to 500 µL with MilliQ water. Subsequently 500 µL of supernatant were removed from the chamber, and replaced with the freshly made H_2O_2/Amplex Red dilute solution. The added solution was rendered homogeneous by gentle pipetting. The final concentrations of H_2O_2 and Amplex Red in the chamber were 40 µM and 10 µM, respectively. Fluorescence microscopy (λ_{ex} = 515 - 560 nm and λ_{em} = 580 nm) was used to monitor the HRP-mediated conversion of the non-fluorescence Amplex Red to the fluorescence resorufin over time.

1.5 Chemical communication within dispersed PTA-CV/Ru_4PCV enzyme/synzyme consortia

FITC-tagged-GOx-filled PTA-CVs (25 µL of a stock dispersion in 500 uL of PBS buffer 0.01 M pH 6.5), RITC-tagged-HRP-filled PTA-CVs (25 µL) and Ru_4PCVs (25 µL) were prepared as described in Methods *FITC-tagged GOx- or RITC-tagged HRP-containing PTA-CVs* and *Preparation of Ru_4PCVs*, washed as described in Methods *Preparation of Ru_4PCVs*, and homogeneously mixed in an Eppendorf tube with PBS buffer (115 µL, 10 mM, pH 6.5) before depositing them on a Petri dish. Substrates glucose (100 µL, 150 mM in PBS 10 mM pH 6.5) and *o*-PD (10 µL, 30 mM in PBS 10 mM pH 6.5) were added, and images were acquired every 15 s. for 1 h. Images were captured simultaneously on four different channels using a fluorescence confocal microscope, as described in Supplementary Table 1.

Supplementary Table 1: Details of the fluorescence confocal microscope settings employed in this study.

Channel 1	*o*-PD	λ_{exc} = 405 nm	λ_{em} = 550-625 nm
Channel 2	FITC	λ_{exc} = 488 nm	λ_{em} = 500-550 nm
Channel 3	RITC	λ_{exc} = 561 nm	λ_{em} = 590-650 nm
Channel 4	Brightfield		

Brightfield images were also acquired to visualize the non-fluorescent Ru_4PCVs. All image stacks were then analysed with ImageJ.

1.6 Determination of PTA-CV/Ru_4PCV Ru_4PCVs enzyme/synzyme activities

A BMG LABTECH CLARIOstar High Performance Monochromator Multimode Microplate Reader was used to determine the PTA-CV/Ru_4PCV activities in the communication/competition experiments described in section 1.5. Fresh samples of FITC-tagged-GOx-filled PTA-CVs, RITC-tagged-HRP-filled PTA-CVs were prepared as described in Methods *FITC-tagged GOx- or RITC-tagged HRP-containing PTA-CVs*, and were washed. A fresh sample of Ru_4PCVs was prepared as described in Methods *Preparation of Ru_4PCVs*, except three batches of Ru_4PCVs were concentrated into 500 µL of PBS buffer (10 mM pH 6.5)

to give a triple concentrate of Ru_4PCV dispersion of 6 mg mL^{-1}, instead of 1.5 mg mL^{-1}. The microplate was prepared by introducing in sequence GOx-filled PTA-CVs (25 µL), HRP-filled PTA-CVs (25 µL), variable volumes of Ru_4PCVs (0, 25, 70 and 140 µL), and variable volumes of PBS buffer (140, 115, 70 and 0, 10 mM pH 6.5). The substrates *o*-PD (10 µL,30 mM in PBS 10 mM, pH 6.5) and glucose (100 µL, 150 mM in PBS 10 mM, pH 6.5) were automatically injected immediately before the measurements. The final solution volume in each well was 300 µL. The *o*-PD solution was prepared immediately before use to minimise degradation from atmospheric oxygen. The kinetics were tracked by fluorescence spectroscopy (λ_{exc} = 405-410 and λ_{em} = 550 nm) by acquiring data points every 2 s for 15 min. The 2,3-DAP concentration over time was then determined by allowing the enzymatic reaction to go to completion and determining the fluorescence intensity when all *o*-PD was converted to product.

1.7 Fabrication and enzyme/synzyme activity of PTA-CV/Ru_4PCV-containing proteinosomes

FITC-tagged-GOx-filled PTA-CVs, RITC-tagged-HRP-filled PTA-CVs and Ru_4PCVs were prepared separately as described in Methods *FITC-tagged GOx- or RITC-tagged HRP-containing PTA-CVs* and *Preparation of Ru_4PCVs*, allowed to sediment to the bottom of an Eppendorf tube, and the supernatant was removed. Subsequently, 10 µL of each PCV population were mixed together in a separate Eppendorf tube. In another Eppendorf tube 15 µL of an aqueous solution of RITC-labelled BSA/PNIPAM nanoconjugates (8 mg/mL) were mixed with 5 µL of glucose solution (0.55 M in PBS buffer 10 mM, pH 6.5) and 5 µL of *o*-PD (11 mM in PBS buffer 10 mM, pH 6.5). This solution was quickly injected into the Eppendorf tube containing the ternary PCV mixture, the dispersion gently mixed, and then injected into a 1.75 mL glass vial containing 1 mL of 2-ethyl-1-hexanol. The vial was shaken for 30 s to obtain PCV-containing proteinosomes as water-in-oil emulsion droplets. The sample was then loaded into a microscope channel slide, which was sealed to avoid evaporation. Images were acquired every 30 s for 2 h using a fluorescence confocal microscope. Images were captured simultaneously on four different channels, as described in Supplementary Table 2.

Supplementary Table 2: Details of the fluorescence confocal microscope settings employed in this study.

Channel 1	*o*-PD	λ_{exc} = 405 nm	λ_{em} = 550-625 nm
Channel 2	FITC	λ_{exc} = 488 nm	λ_{em} = 500-550 nm
Channel 3	RITC	λ_{exc} = 561 nm	λ_{em} = 590-650 nm
Channel 4	Brightfield		

Brightfield images were also acquired to visualize the non-fluorescent Ru_4PCVs. All images were then analysed with ImageJ.

2. Supplementary Figures

a) b)

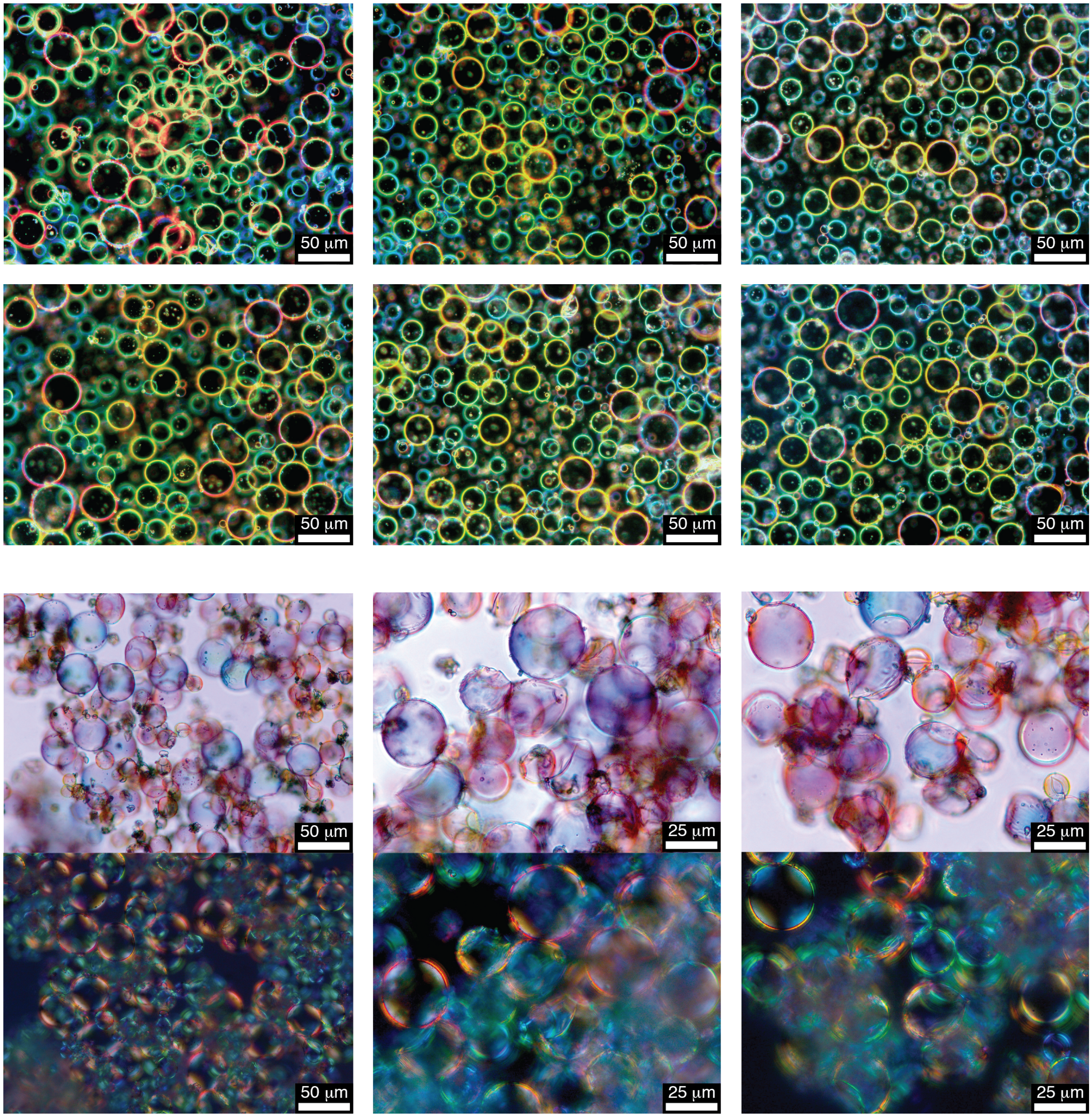

Supplementary Figure 1: Optical microscopy images of Ru_4PCVs. a, Dark field microscopy images of different batches of washed Ru_4PCVs in PBS buffer 0.01 M pH 6.5 showing the fidelity of the preparation procedure. **b**, Bright field images of a lyophilized sample of Ru_4PCVs mounted on a microscope slide (top), and corresponding images acquired using a cross-polarized filter (bottom) showing strong birefringence due to formation of the Ru_4POM/PTA membrane.

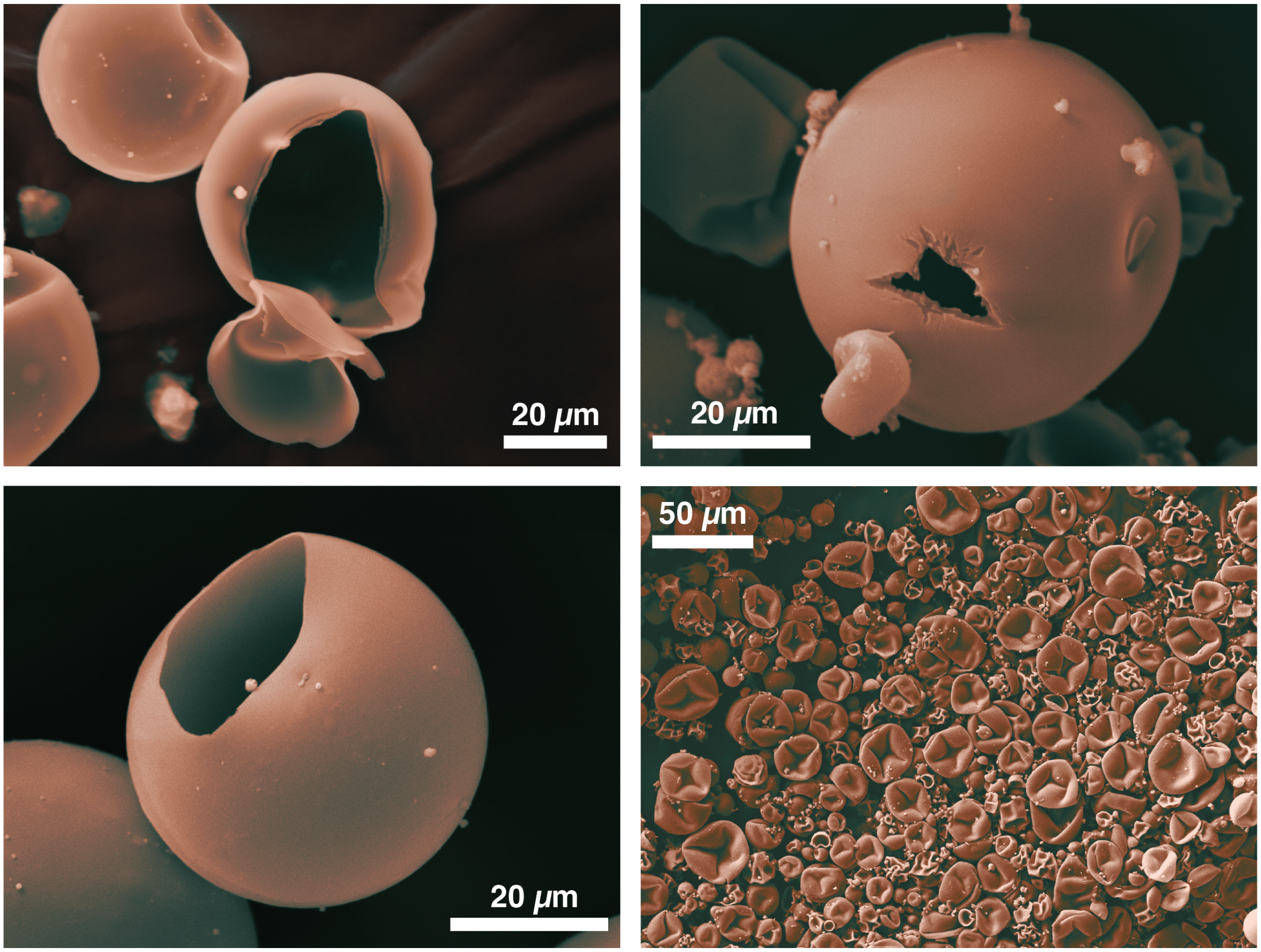

Supplementary Figure 2: SEM images of Ru_4PCVs. Micrographs show lyophilised samples. Bottom-right image shows Ru_4PCVs that have been compressed with a spatula, highlighting the pliant behaviour of the PDDA/POM membrane. Images have been artificially coloured to mimic the real tinge of the material.

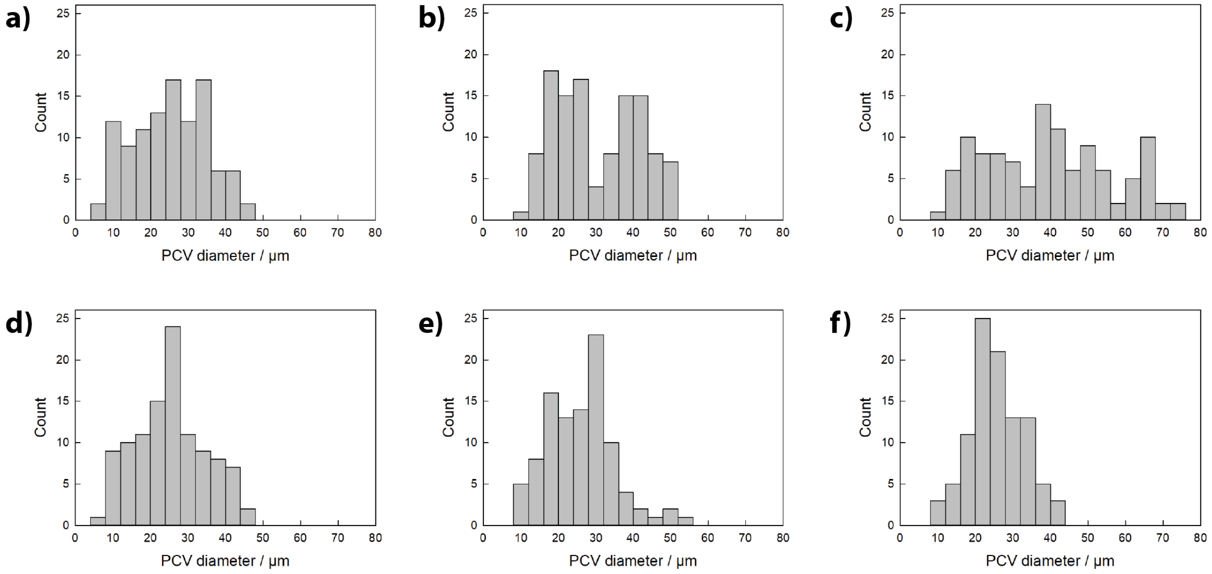

Supplementary Figure 3: Effect of stirring on the Ru_4PCV size distribution. a-c, Size distribution of Ru_4PCVs prepared using different stirring times for the precursor PDDA/ATP coacervate phase before addition of Ru_4POM/PTA: from left to right 30, 60 and 120 s, respectively. **d-f**, Size distribution of Ru_4PCVs synthesized using different stirring times after the addition of the Ru_4POM/PTA mixture: from left to right 10, 30, and 120 s, respectively. Increase in stirring time for the PDDA/ATP coacervate phase before addition produce an increase in the mean PCV diameter and a progressively wider size distribution. In contrast, increasing the stirring times after addition of the Ru_4POM/PTA mixture only has a marginal effect on the PCV mean diameter and size distribution.

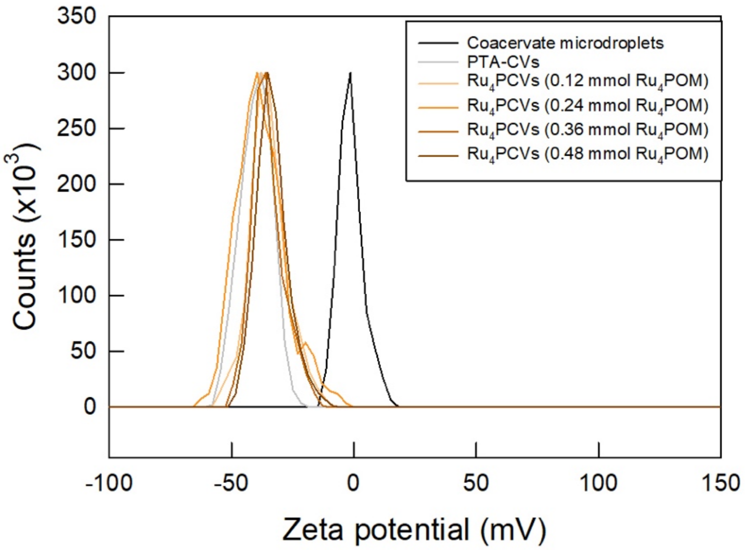

Supplementary Figure 4: Zeta-potential of Ru_4PCVs as a function of Ru_4POM concentration in the POM mixture. PCVs were prepared by the same procedure, but the volume of Ru_4POM stock solution was varied to prepare POM mixtures containing 0.12, 0.24, 0.36, and 0.48 mmol of Ru_4POM. No significant change in the surface charge of the Ru_4PCVs is observed. In each case, a marked decrease in the PCV surface charge from *ca.* 0 mV (coacervate micro-droplets) to -35.6 ± 1.4 mV (Ru_4PCVs) and -39.8 ± 0.7 mV (PTA-CVs) is observed after the coacervate-to-vesicle transition.

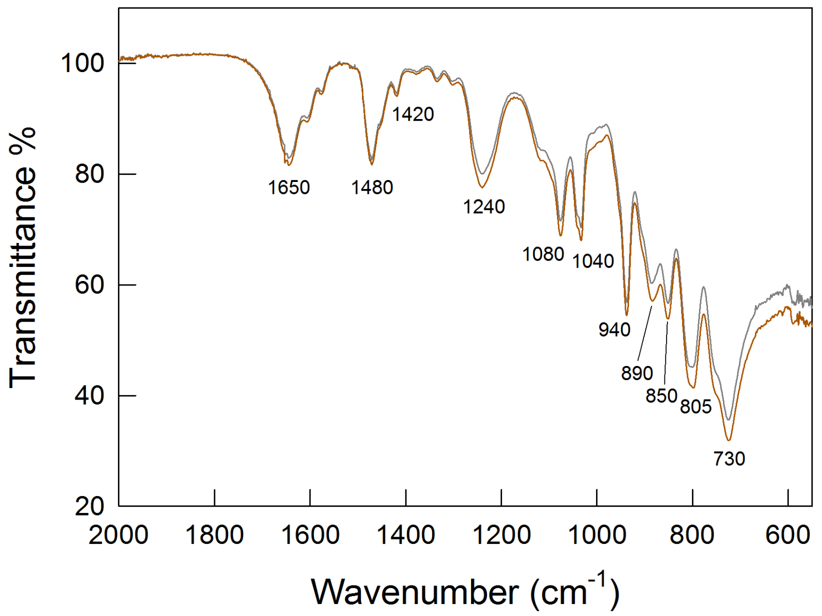

Supplementary Figure 5: FT-IR spectroscopy characterization of Ru_4PCVs. FT-IR spectra of lyophilized samples of PTA-CVs (grey plot) and Ru_4PCVs (light brown plot) acquired on lyophilized powders. The FT-IR spectra are very similar and show the typical stretching modes of PDDA (2950 cm^{-1} C-H stretching; 1640 cm^{-1} C=C stretching – impurity; 1480 cm^{-1} in plane bending C-H alkene – impurity)[2,3], ATP (1650 cm^{-1} scissoring of $–NH_2$; 1510 cm^{-1} stretching C_8-N; 1420 cm^{-1} stretching N_1-C_6-N_6; 1240 cm^{-1} stretching N_7-C_8-N_9; 1110 cm^{-1}: stretching C-O)[4], and POM (940 cm^{-1} W=O stretching; 850 cm^{-1} W-O-W stretching of corner-sharing octahedral; 805-730 cm^{-1} W-O-W stretching of corner-sharing octahedral)[5].

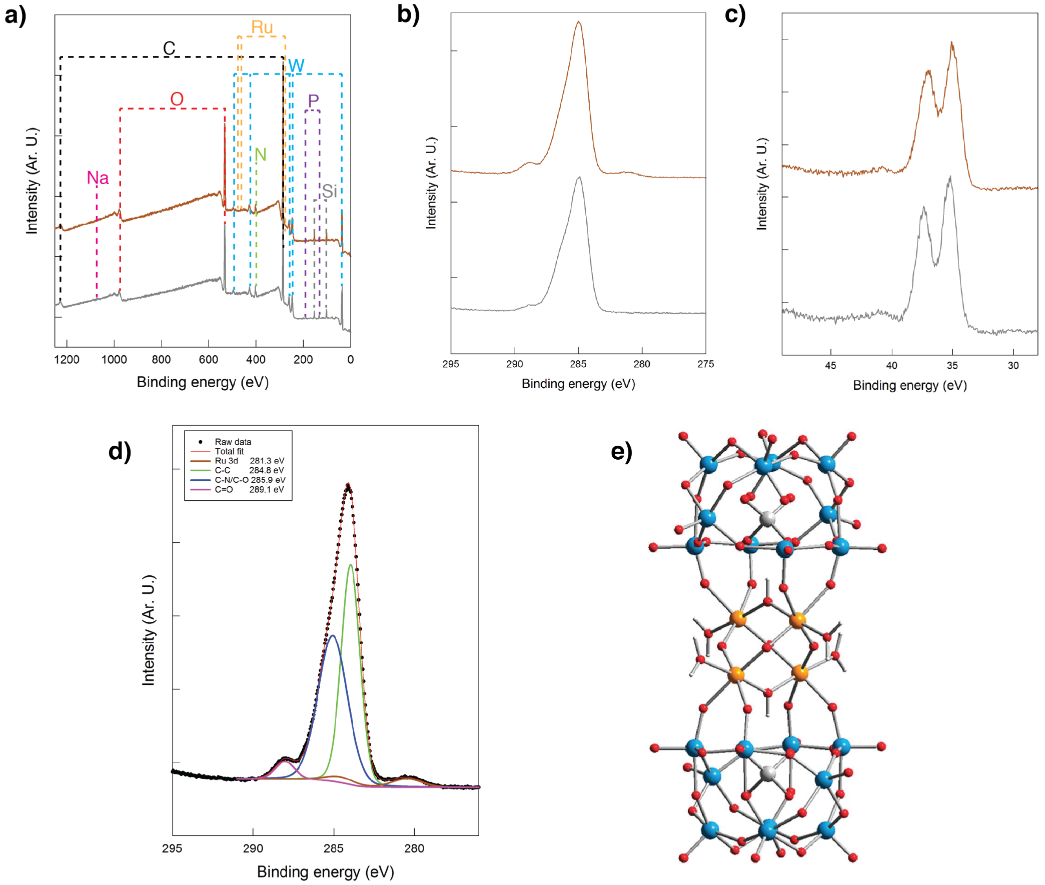

Supplementary Figure 6: XPS characterization of Ru_4PCVs. **a**, XPS survey comparing the main photoemission lines detected for Ru_4PCVs (light brown spectrum) and PTA-CVs (grey spectrum). The photoemission lines of the main elements are labelled in the graph. **b**, Comparison between the high-resolution scans of the C 1s region for Ru_4PCVs (light brown spectrum) and PTA-CVs (grey spectrum). A shoulder peak at 281.3 eV in the Ru_4PCV spectrum is due to the Ru $3d_{5/2}$ photoemission line, which is absent from the corresponding spectrum of PTA-CVs. **c**, Comparison between the high-resolution scans of the W 4f photoemission lines for Ru_4PCVs (light brown spectrum) and PTA-CVs (grey spectrum), highlighting a prevalence of W(VI) in both samples (peaks at 37.1 and 35.0 eV, W $4f_{5/2}$ and W $4f_{7/2}$, respectively). **d**, Deconvolution of the C 1s and Ru 3d peaks for the Ru_4PCV sample. The deconvolution highlights the presence of carbon atoms belonging to different chemical environments: 285.9 eV C–N and C–O from ATP and PDDA (blue curve), 284.8 eV C–C from ATP and PDDA (green curve). Two typical peaks for the Ru 3d photoemission line at 285.5 eV (Ru $3d_{3/2}$) and 281.3 eV (Ru $3d_{5/2}$) are shown in light brown The detected binding energies are typical of a Ru (IV) species[6], indicating that the Ru_4POM did not decompose when complexed with the PDDA/ATP coacervate micro-droplet to form the PCV. **e**, Three-dimensional molecular model of Ru_4POM.[7] Yellow: ruthenium atoms; grey: silicon atoms; blue: tungsten atoms; red: oxygen atoms; white: hydrogen atoms (hydrogens are omitted from γ-SiW_{10} units). Analysis of the relative composition of W and Ru, after normalization of the individual photoemission lines by their sensitivity factor and inelastic mean-free path, gave a W/Ru atomic ratio of 10 ± 4, which agrees within experimental error with the ICP quantitative analysis. The high uncertainty on the measurement is due the attenuation of the Ru signal exerted by the two γ-SiW_{10} units, see (**e**).

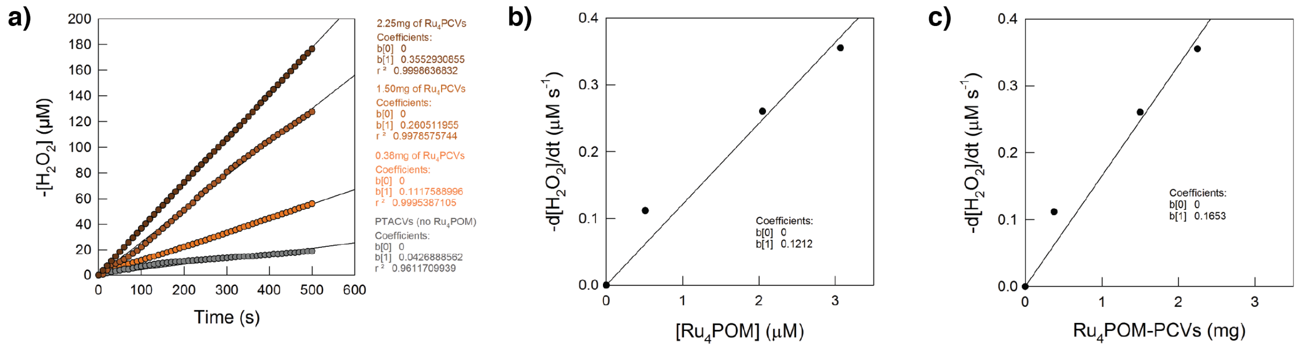

Supplementary Figure 7: Kinetics of Ru_4PCV-mediated hydrogen peroxide decomposition. **a**, Rate constants for the Ru_4POM-catalysed reaction of H_2O_2 decomposition determined using different amounts of Ru_4PCVs: 2.25 mg (dark brown plot), 1.50 mg (brown plot), 0.38 mg (light brown plot), and 0 mg (grey plot). **b,c**, Plots of reaction rate (*R*) (µM s^{-1}) against Ru_4POM concentration (µM or mg) used to prepare the Ru_4PCVs (initial concentration of H_2O_2, 2.8 M).

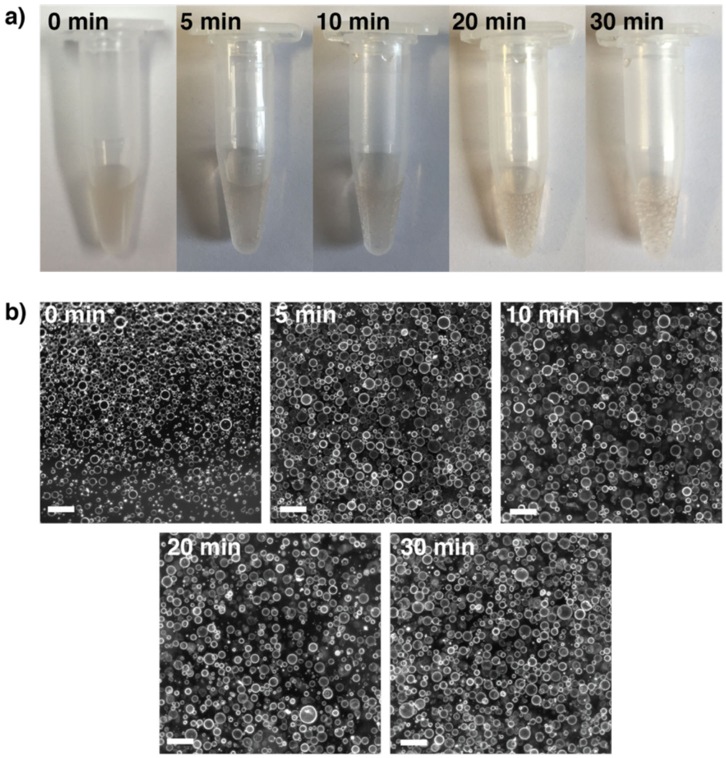

Supplementary Figure 8: Stability of Ru_4PCVs under catalytic conditions. **a**) A batch of Ru_4PCVs (0.38 mg Ru_4PCVs, 250 µL PBS buffer (10 mM, pH 6.5)) was placed in an Eppendorf tube and 100 µL of a H_2O_2 solution (30 w/w %) then added. Photographs of the Eppendorf tube were acquired after 0, 5, 10, 20 and 30 min after initiation of the catalytic reaction. The images show progressive formation of dioxygen bubbles in the solution as a result of the protocell-mediated catalytic process. **b**) Optical microscopy images of the corresponding Ru_4PCVs shown in (**a**) during the catalytic reaction; scale bars; 100 µm. The images show that the Ru_4PCV are stable under the experimental conditions employed and do not burst or disassemble as a result of dioxygen production. We attribute this to the incorporation of Ru_4POM specifically within the POM/PDDA outer membrane.

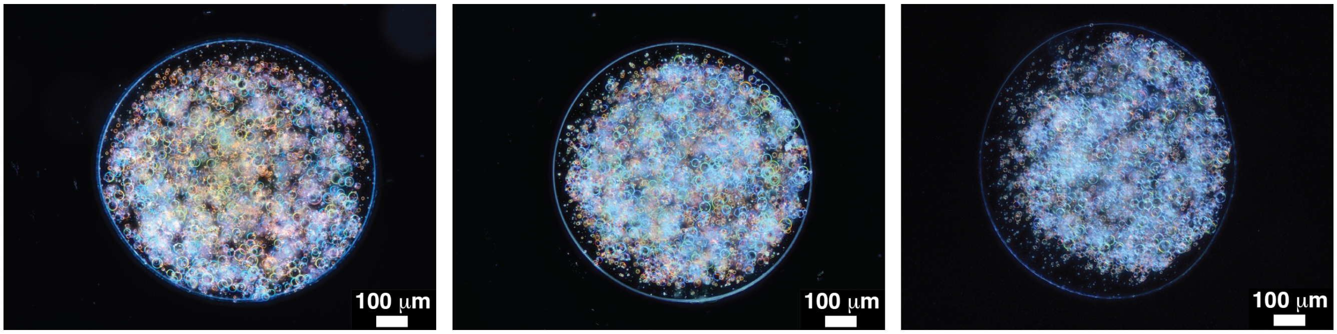

Supplementary Figure 9: Ru_4PCV-containing aminoclay/DNA synthetic protocells. Dark field microscopy images recorded in water showing an outer aminoclay/DNA membrane enclosing a dense population of catalytically active Ru_4PCVs.

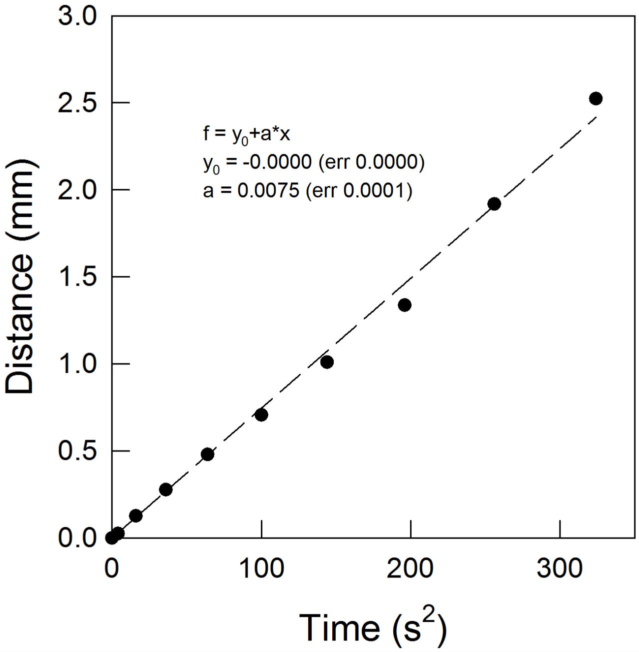

Supplementary Figure 10: Oxygen bubble-dependent buoyancy in Ru_4PCV-containing aminoclay/DNA protocells. Plot shows distance travelled by the protocell (see Video 4) over time-squared. The protocell acceleration (7.5 ± 0.1 μm s^{-2}) was calculated by the linear fitting slope.

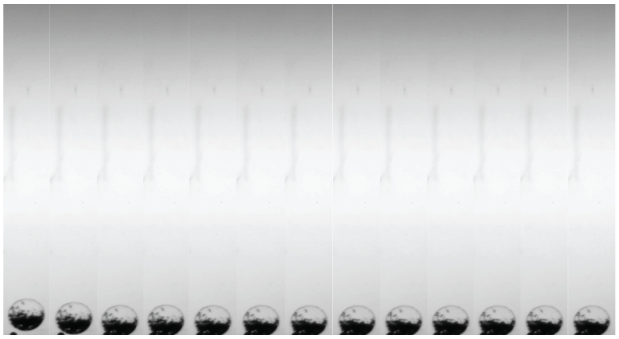

Supplementary Figure 11: Control buoyancy experiment. Composite image (see Video 4) showing a single PTA-CV-filled aminoclay/DNA microcapsule (no Ru_4POM) located at the bottom of a water-filled cuvette containing a gradient of H_2O_2 (higher concentration at the bottom). No buoyancy is observed. PTA-CVs are observed as dark material inside the aminoclay/DNA capsule. Frames displayed at intervals of 1.2 s.

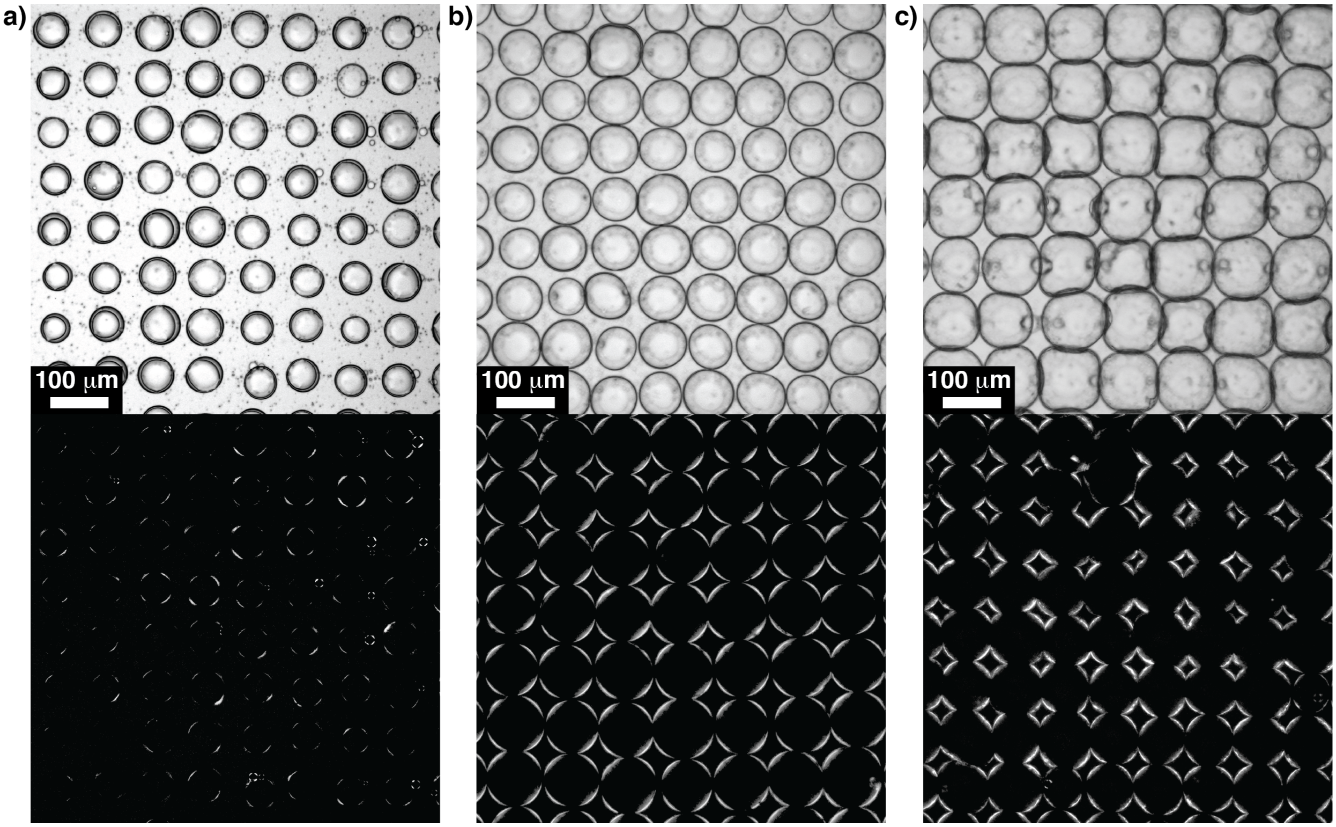

Supplementary Figure 12: Formation of acoustically patterned arrays of Ru_4PCVs in a unidirectional PTA/Ru_4POM chemical gradient. Corresponding brightfield (top) and cross-polarized (bottom) microscopy images showing three different domains observed within the same 2D square microarray grid of immobilized PDDA/ATP droplets 30 minutes after injection of a PTA/Ru_4POM mixture at one side of the sample chamber (left side in the images). **a**, Non-contact small-sized Ru_4PCVs (mean diameter, 77 ± 6 μm; membrane-to-membrane separation, 38 ± 7 μm); **b**, Medium-sized Ru_4PCVs (mean diameter, 108 ± 8 μm) with membrane-membrane contacts; **c**, Large-sized Ru_4PCVs (mean diameter, 114 ± 8 μm) showing compression-induced deformation. In each case, the graded protocells show birefringent PDDA/POM membranes, consistent with the presence of an enclosing POM/PDDA membrane.

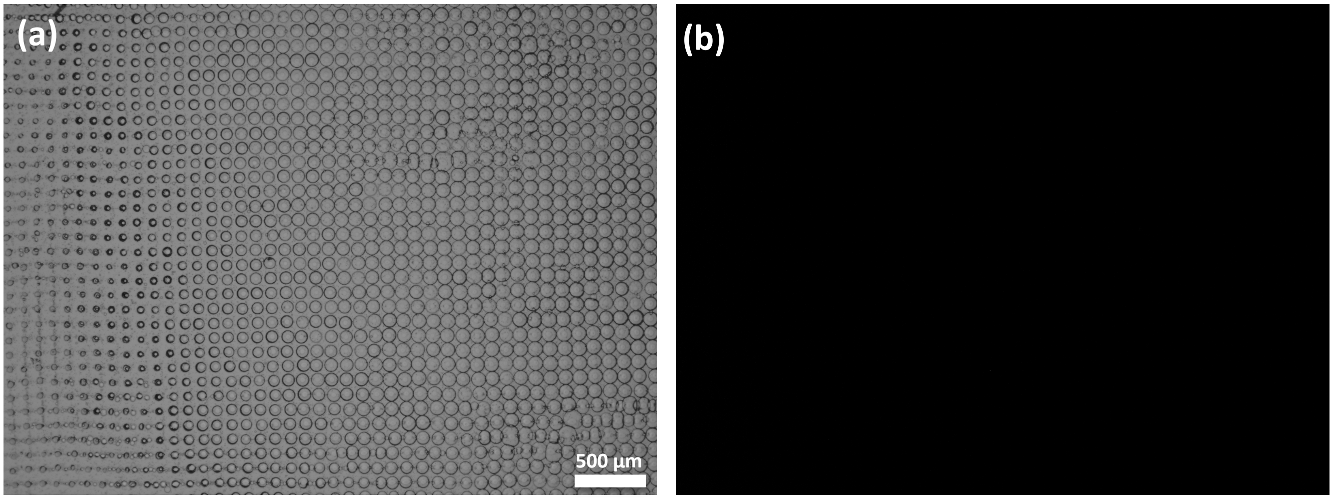

Supplementary Figure 13: Control peroxidation experiment. **a**, Optical microscopy and **b** red fluorescence microscopy images showing an acoustically patterned array of Ru_4PCVs (no encapsulated HRP) after addition of Amplex Red and H_2O_2. The absence of red fluorescence in **b** indicates that the synzyme-based protocells are inactive with regard to Amplex Red peroxidation.

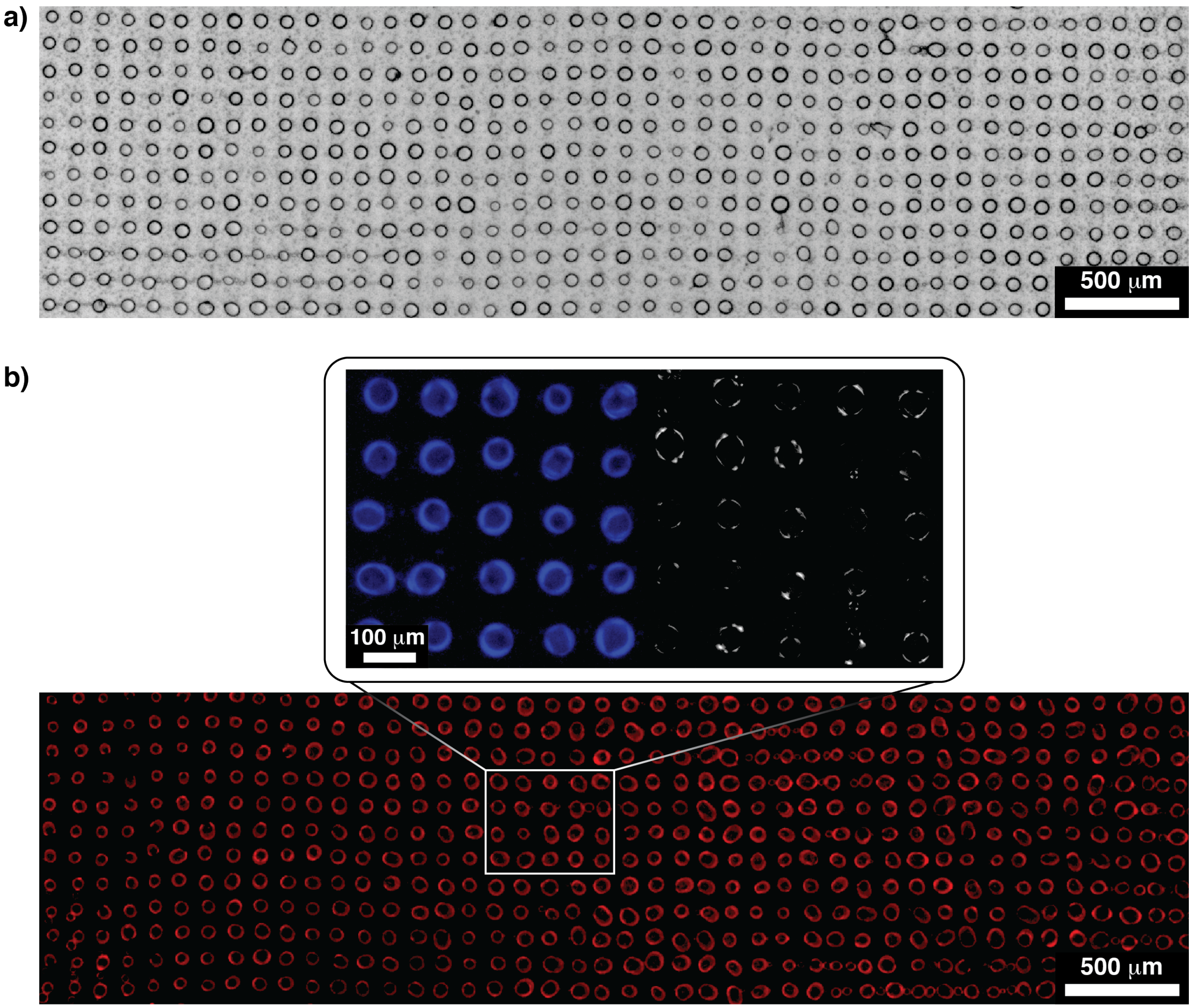

Supplementary Figure 14: Catalytic reactions in HRP-containing PTA-CV arrays. a, Brightfield microscopy image showing an acoustically trapped 2D array of immobilized HRP-containing PDDA/ATP coacervate microdroplets. The field of view is 5.0 x 1.5 mm. **b**, Fluorescence microscopy images of an array of HRP-containing PTA-CVs 16 min after homogeneous addition of H_2O_2 and Amplex Red (final concentrations 40 and 10 μM, respectively). Red fluorescence associated with the HRP-mediated production of resorufin in the PTA-CVs is approximately uniform throughout the micro-array (bottom image). The enlarged insets show localized blue fluorescence associated with Dylight405-labelled HRP (left side) and membrane-induced birefringence (right) confirming retention of the functional enzyme within the PTA-CVs. The experiment demonstrates that in the absence of Ru_4POM, the array displays isotropic catalytic properties rather than the graded functionality observed when both peroxidase and catalase-like pathways are present.

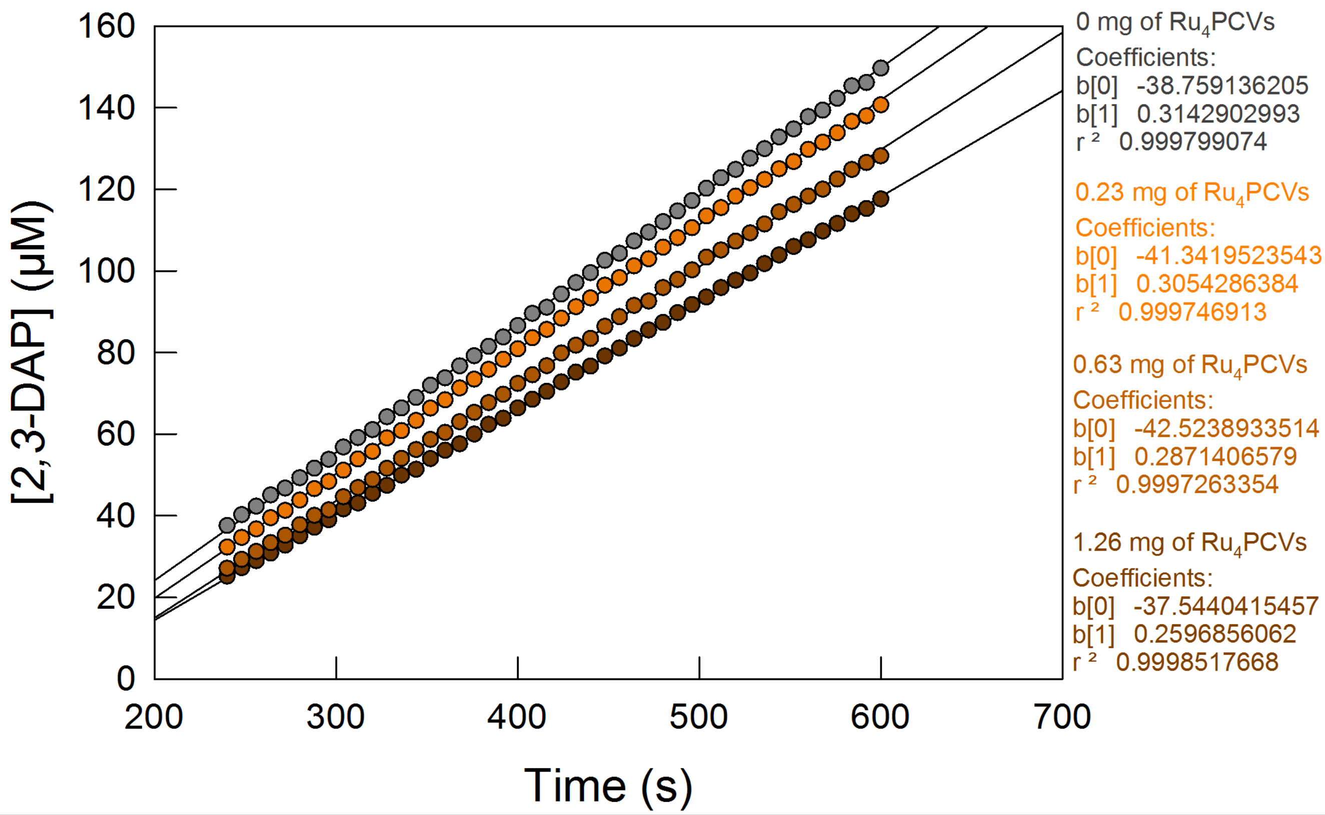

Ru_4PCVs (mg)	HRP-PCVs (mg)	Ru_4CVs : HRP-PCVs	Ru_4PCVs v_0 (μM min^{-1})	HRP-PCVs v_0 (μM min^{-1})
0	0.08	0	0.0	18.9
0.23	0.08	3	0.5	18.4
0.63	0.08	8	1.7	17.2
1.26	0.08	16	3.3	15.6

Supplementary Figure 15: Chemical communication within dispersed PTA-CV/Ru_4PCV enzyme/synzyme consortia. Determination of the interactivity within a ternary population of GOx-containing PTA-CVs, HRP-containing PTA-CVs and Ru_4PCVs. (**Top**) Graph showing the linear region of the plots of 2,3-DAP concentration *versus* time that were used to determine the linear fittings shown in the Table (**bottom**).

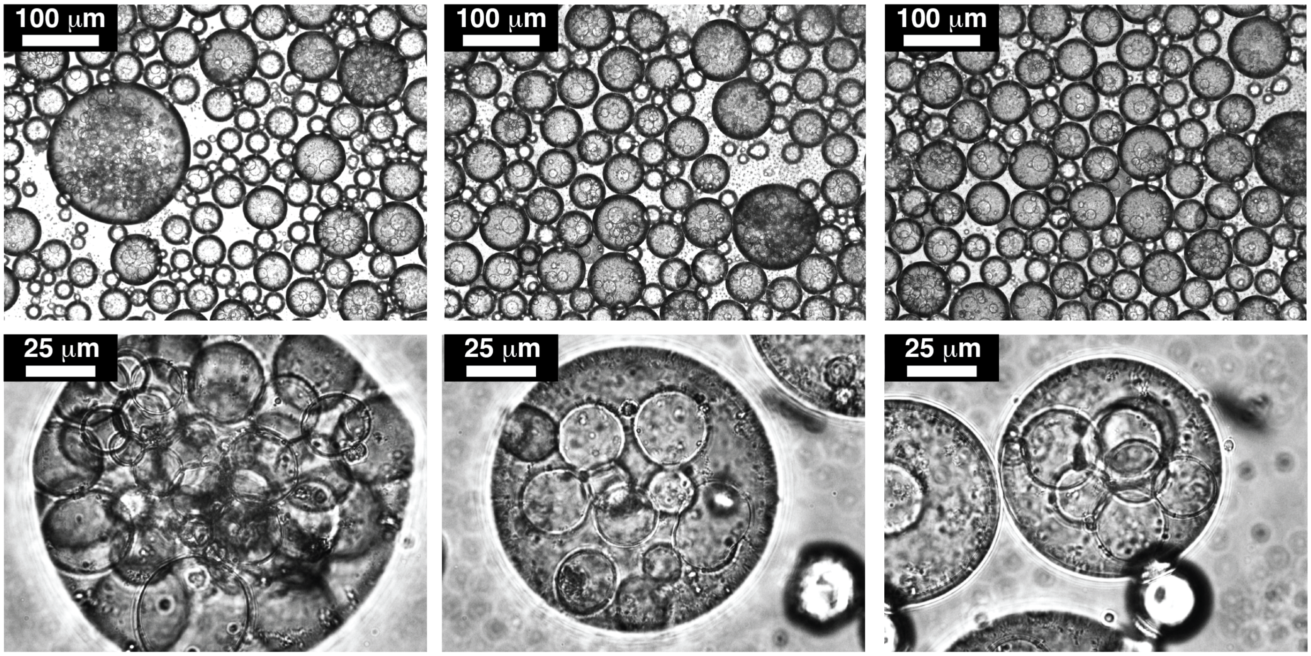

Supplementary Figure 16: Multi-compartmentalized PTA-CV/Ru_4PCV-containing water-in-oil emulsions. Brightfield microscopy images of water droplets-in-oil consisting of an interfacial protein-polymer (BSA/PNIPAM) monolayer membrane and internalized ternary protocell population of GOx-containing PTA-CVs, HRP-containing PTA-CVs and Ru_4PCVs. As a consequence, parallel catalytic reactions involving enzyme- and synzyme-active protocells are established within the host micro-compartment.

3. Supplementary References

1 Sartorel, A. *et al.* Oxygenic polyoxometalates: a new class of molecular propellers. *Chem. Commun.* **47**, 1716-1718, (2011).

2 Mwangi, I. W., Ngila, J. C., Ndungu, P. & Msagati, T. A. M. Method Development for the Determination of Diallyldimethylammonium Chloride at Trace Levels by Epoxidation Process. *Water Air Soil Pollut.* **224**, (2013).

3 Yang, D. Q., Rochette, J. F. & Sacher, E. Spectroscopic evidence for pi-pi interaction between poly(diallyl dimethylammonium) chloride and multiwalled carbon nanotubes. *J. Phys. Chem. B* **109**, 4481-4484, (2005).

4 Tajmirriahi, H. A. & Theophanides, T. Adenosine-5'-monophosphate complexes of pt(ii) and mg(ii) metal-ions - synthesis, ft-ir spectra and structural studies. *Inorganica Chim. Acta - Bioinorg. Chem.* **80**, 183-190, (1983).

5 Shringarpure, P. A. & Patel, A. Supported dodecaphosphotungstate and undecaphosphotungstate: a study on the kinetic behavior for the oxidation of styrene. *React. Kinet. Mech. Catal.* **103**, 165-180, (2011).

6 Morgan, D. J. Resolving ruthenium: XPS studies of common ruthenium materials. *Surf. Interface Anal.* **47**, 1072-1079, (2015).

7 Sartorel, A. *et al.* Polyoxometalate embedding of a tetraruthenium(IV)-oxo-core by template-directed metalation of gamma-SIW10O36 (8-): A totally inorganic oxygen-evolving catalyst. *J. Am. Chem. Soc.* **130**, 5006-5007, (2008).
