# Supplementary material for: Catalytic processing in ruthenium-based polyoxometalate coacervate protocells
Source: Nat Commun. 2020 Jan 3;11:41. doi: 10.1038/s41467-019-13759-1 (PMC6941959; doi:10.1038/s41467-019-13759-1)
Supplement: Supplementary file 2 — Description of Additional Supplementary Files [file 41467_2019_13759_MOESM2_ESM.docx]

**Description of Additional Supplementary Files**

File name: Supplementary Movie 1
Description: Oxygen bubble-dependent buoyancy in Ru_4_PCV-containing aminoclay/DNA protocells. The video compares two experiments. On the left it shows a control experiment where a single PTA-CV-filled aminoclay/DNA multi-compartmentalized microcapsule is allowed to sink under gravity in a cuvette containing an increasing concentration of H_2_O_2_. Because the synthetic protocell is not chemically programmed to react to the oxidative environment, it simply sinks and sediments at the bottom of the cuvette (where there is the highest concentration of H_2_O_2_). In contrast, on the right the video shows a Ru_4_PCV-filled aminoclay/DNA synthetic protocell that is allowed to sink under gravity in a cuvette containing an increasing concentration of H_2_O_2_. Due to the ability of the Ru_4_PCV proto-organelles to rapidly react with H_2_O_2_ to produce water and molecular oxygen, an oxygen bubble (dark expanding spot) is nucleated within the host-guest protocell. The increase in buoyancy enables the multi-compartmentalized capsule to escape the “toxic” H_2_O_2_-rich environment present at the bottom of the cuvette. Both videos are played at double speed.

File name: Supplementary Movie 2
Description: Preparation of 2D Ru_4_PCV micro-arrays by acoustic standing wave patterning. The video shows a 7x7 array of non-catalytic membrane-free coacervate microdroplets undergoing reconfiguration to catalytic membrane-bounded sub-divided vesicles. The real time of the video is 30 min with individual frames acquired every 10 sec.

File name: Supplementary Movie 3
Description: Chemical communication within dispersed PTA-CV/Ru_4_PCV enzyme/synzyme consortia. The video is recorded from a dispersed PCV community comprising a ternary population of FITC-tagged GOx-containing PTA-CVs (green fluorescence), FITC-tagged HRP-containing PTA-CVs (red fluorescence) and Ru_4_PCVs (untagged). Upon addition of glucose and *o*-PD (50 mM and 1 mM, respectively), the video shows three GOx-containing PCVs that generate H_2_O_2_, which acts as a diffusive signal for the production of 2,3-DAP (green fluorescence) in the single HRP-containing PTA-PCV. In contrast, the adjacent single Ru_4_PCV decomposes the H_2_O_2_ signal to water and molecular oxygen. The video shows a progressive increase of green fluorescence initially in HRP-PCV and then in the other types of protocells due to diffusion of 2,3-DAP into the medium. Oxygen bubbles can be occasionally observed emanating specifically from the untagged Ru_4_PCV. The real time length of the video is 67 min with individual frames acquired every 15 s. This video was used to generate data shown in Figure 4c.

File name: Supplementary Movie 4
Description: Enzyme/synzyme activity of PTA-CV/Ru_4_PCV-containing proteinosomes. The video shows a ternary population of FITC-tagged GOx-containing PTA-CVs (green fluorescence), RITC-tagged HRP-containing PTA-CVs (red fluorescence), and Ru_4_PCVs (untagged) enclosed within a RITC-labelled BSA/PNIPAM proteinosome membrane. The multi-compartmentalized system is prepared as a water droplet-in-oil emulsion. Due to the presence of glucose and *o*-PD substrates in the water phase (50 mM and 1 mM, respectively) the GOx-containing PCVs generate H_2_O_2_, which acts as a diffusive signal for the production of 2,3-DAP (green fluorescence) in the HRP-containing PCVs. In contrast, the Ru_4_PCV population decomposes the H_2_O_2_ signal to water and molecular oxygen. The video shows a progressive increase of green fluorescence initially in HRP-PCVs and then in the other populations and in the bulk oil phase due to diffusion of 2,3-DAP. The real time length of the video is 123 min with individual frames acquired every 30 s.
